# Innate Immune Sensing of Viruses and Its Consequences for the Central Nervous System

**DOI:** 10.3390/v13020170

**Published:** 2021-01-23

**Authors:** Hina Singh, Jeffrey Koury, Marcus Kaul

**Affiliations:** 1 Division of Biomedical Sciences, School of Medicine, University of California, Riverside, CA 92521, USA; hina.singh@medsch.ucr.edu (H.S.); jeffrey.koury@medsch.ucr.edu (J.K.); 2 Infectious and Inflammatory Disease Center, Sanford Burnham Prebys Medical Discovery Institute, 10901 North Torrey Pines Road, La Jolla, CA 92037, USA

**Keywords:** toll-like receptor, C-type lectin receptors, RIG-I-like receptors, NOD-like receptors, inflammasome, interferons, cytokines, signaling, central nervous system

## Abstract

Viral infections remain a global public health concern and cause a severe societal and economic burden. At the organismal level, the innate immune system is essential for the detection of viruses and constitutes the first line of defense. Viral components are sensed by host pattern recognition receptors (PRRs). PRRs can be further classified based on their localization into Toll-like receptors (TLRs), C-type lectin receptors (CLR), retinoic acid-inducible gene-I (RIG-I)-like receptors (RLRs), NOD-like receptors (NLRs) and cytosolic DNA sensors (CDS). TLR and RLR signaling results in production of type I interferons (IFNα and -β) and pro-inflammatory cytokines in a cell-specific manner, whereas NLR signaling leads to the production of interleukin-1 family proteins. On the other hand, CLRs are capable of sensing glycans present in viral pathogens, which can induce phagocytic, endocytic, antimicrobial, and pro- inflammatory responses. Peripheral immune sensing of viruses and the ensuing cytokine response can significantly affect the central nervous system (CNS). But viruses can also directly enter the CNS via a multitude of routes, such as the nasal epithelium, along nerve fibers connecting to the periphery and as cargo of infiltrating infected cells passing through the blood brain barrier, triggering innate immune sensing and cytokine responses directly in the CNS. Here, we review mechanisms of viral immune sensing and currently recognized consequences for the CNS of innate immune responses to viruses.

## 1. Introduction

Viruses are infectious agents that continue to pose a threat to public health. Advances in medical research have resulted in vaccinations, which can provide protective immunity against viruses, antiviral drugs, which can interfere with viral replication, and improvements in diagnostics. However, many viruses, such as influenza virus and human immunodeficiency virus (HIV), undergo genetic mutations thereby rendering vaccines ineffective and/or developing resistance to antiviral drugs [1,2]. Uncontrolled propagation of these mutated viruses can lead to epidemics/pandemics as seen with the 1918 influenza, AIDS, Ebola virus, dengue, Zika and most recently with the emergence of the novel severe acute respiratory syndrome coronavirus 2 (SARS-CoV-2) in 2019 [2].

Modern day ease of transportation and globalization can facilitate the spread of pathogens worldwide. In addition, with increasing global populations and urbanization, people are more likely to live in densely populated areas resulting in much faster spread. Several other factors including poverty and climate change can contribute to outbreaks. Bioterrorism is another serious threat that human society is facing. It becomes a serious challenge when the viral disease is hard to treat and/or highly contagious; potentially resulting in public health disaster. Beyond the global health concerns, viruses can also cause severe societal and economic damage. Although, several attempts have been made to develop a universal vaccine against viruses like influenza and Ebola virus in order to provide immunity against all the respective subtypes, those efforts have until now either failed for unknown reasons or are still in clinical trials [3,4].

Considering this situation, it is important to develop novel strategies which can help us prevent the deleterious effects of these deadly viruses. To accomplish this goal, it is of utmost importance for us to elucidate virus-host interactions, pathological viral mechanisms, antiviral responses, and host innate and adaptive immune responses. Many viruses can cause life-threatening complications, if they enter the central nervous system (CNS), which can also serve as a reservoir for a plethora of viruses. In this review we discuss recent progress in the understanding of the host innate immune system’s sensing of viruses, as well as the sometimes-pathogenic consequences of innate immune sensing in the CNS. Since the processes of viral sensing remain largely conserved between the periphery and the CNS, we discuss them mostly together before going more specifically into consequences for the CNS. Expression levels of related host genes and robustness of the responses may vary between peripheral cells (i.e., macrophages (Mɸ), dendritic cells (DCs)) and CNS cells (i.e., microglia, neurons, astrocytes) for reasons inherent to tissue location but also due to viral tropism.

## 2. Viral Recognition and Antiviral Response

Recognition of viral antigens plays a critical role in nonspecific or innate immune host resistance. The recognition strategy of the host is based on the detection of conserved molecular structures that occur in patterns and are the essential product of viral physiology. These structures are known as pathogen-associated molecular patterns (PAMPs). The PAMPs are unique to viruses and other microorganism and not produced by host cells. Different types of molecular structures can act as microbial PAMPs, such as lipopolysaccharide (LPS), endotoxins, peptidoglycan, flagellin, lipoteichoic acid, double stranded RNA (dsRNA), single stranded RNA (ssRNA), DNA, CpG DNA and nucleic acid motifs [5].

These PAMPs are recognized by germline-encoded pattern recognition receptor (PRRs) of the infectious agent’s host. PRRs are proteins expressed by a variety of cell types including fibroblasts, epithelial cells and innate immune cells like DCs, Mɸ, monocytes, neutrophils and lymphocytes of the adaptive immune system [6]. PRRs can be further classified based on their localization, ligand specificity and functions. Based on localization PRRs can be divided into (i) membrane bound PRRs, such as Toll-like receptors (TLRs) and C-type lectin receptors (CLRs), and (ii) cytoplasmic PRRs, including retinoic acid inducible gene-I (RIG-I)-like receptors (RLRs), nucleotide-binding oligomerization domain (NOD)-like receptors, and cytosolic DNA sensors (CDS), such as cyclic GMP-AMP synthetase [7].

Upon viral infection, virus-associated nucleic acids, such as DNA and RNA, or dsRNA produced by infected cells can be sensed by PRRs [1]. After recognition, the appropriate PRR initiates various host defense pathways leading to the production of proinflammatory cytokines/chemokines, type I interferons (IFNs) and antimicrobial proteins [1,8]. Type I IFN signaling by both IFNβ and multiple IFNα isoforms is important for antiviral responses, as they activate transcription of IFN-inducible genes, which are involved in eliminating viral components from infected cells, inducing apoptosis of infected cells and conferring resistance to viral infection in uninfected cells. The IFNs and the induced, secreted cytokines and chemokines also play an essential role in adaptive and memory immune response [9,10].

## 3. Toll-Like Receptors

Toll-like receptors (TLRs) are the first identified class of PRR and are important in recognizing viral infection and playing an integral role in the innate immune system. TLRs are transmembrane proteins usually expressed on antigen presenting cells like Mɸ and DCs. The TLR family consists of 10 members in humans (TLR1–10), and 12 in mice (TLR1–TLR9, TLR11–TLR13) [11]. TLRs are located in the plasma membrane or on intracellular compartments, such as endoplasmic reticulum (ER), endosome, lysosome and endolysosome. Each TLR subtype is characterized by an extracellular domain with leucine-rich repeats (LRRs) that mediate PAMPs recognition, a transmembrane domain, and a cytoplasmic Toll/IL-1 receptor (TIR) domain that initiates downstream signaling. TLR1, TLR2, TLR4, TLR5, TLR6, and TLR10 are located mainly in the plasma membrane where they interact with viral/microbial pathogens that come in contact with the cell. In contrast, TLR3, TLR7, TLR8, TLR9, TLR11, TLR12, and TLR13 are found intracellularly on endosomal and endolysosomal membranes [12].

### 3.1. Recognition of Viruses by TLRs

Of the 10 TLRs found in humans, TLR2, TLR3, TLR4, TLR7, TLR8, TLR9 and TLR10 are involved in viral recognition. The cell surface TLR2 and TLR4 are involved in recognition of viral envelope proteins on the cell surface. Additionally, TLR2 and TLR4 also play a critical role in recognition of bacterial components like LPS, peptidoglycans and lipoproteins. TLR2 is expressed in variety of cells including monocytes, Mɸ, DCs, B-cells and T-cells. TLR2 senses cytomegalovirus (CMV) via viral envelope glycoprotein B and H, and HCV via core protein [13,14]. Hepatitis B virus (HBV) activates TLR2 signaling in primary human hepatocytes, leading to an anti-HBV immune response [15]. Herpes simplex virus (HSV) is sensed by TLR2 via glycoprotein gH/gl and gB, which is sufficient to activate a nuclear factor kappa B (NF-κB) response [16]. Moreover, another report indicates that HSV-1 interaction with TLR2 can result in lethal encephalitis [17]. Furthermore, TLR2 mediates innate immune response against Junin virus (JUNV) in mice and plays a critical role in viral clearance of Candid 1 (JUNV C1; the vaccine strain of Junín virus) [18].

TLR4 is expressed on many cell types for example, antigen presenting cells, endothelial cells, and thyroid cells. TLR4 senses vesicular stomatitis virus (VSV) via glycoprotein G [19], Ebola virus (EBOV) via glycoprotein [20], and envelope proteins of murine retroviruses MMTV (mouse mammary tumor virus), and MMLV (Moloney murine leukemia virus) [21]. HIV-1 envelope protein gp120 signaling through TLR4 modulates immune activation in human Mɸ [22]. In vitro studies revealed that cells lacking TLR4 are more susceptible to Kaposi sarcoma herpesvirus (KSHV) infection, whereas activation of TLR4 protects cells from infection [23]. In contrast, TLR4 signaling appears to promote MMTV replication in mice and upregulates the expression of MMTV entry receptor on DCs [24,25]. On the other hand, TLR4-deficient (TLR4^−/−^) mice were found to be resistant to H5N1 avian influenza infection [26]. In line with this, others reported TLR4^−/−^ mice were protected from lethal infection by H1N1 influenza [27]. Moreover, both TLR2 and TLR4 are implicated in recognition of damage-associated molecular patterns (DAMPs) released from necrosis-causing infections, including heat shock proteins (HSPs), high mobility group box-1 (HMGB1) protein, and oxidized phospholipids [28]. All these factors can also be released during viral infections that are associated with cell death [26,29,30].

TLR3 recognizes viral dsRNA, small interfering RNAs (siRNAs), and self-RNAs derived from damaged cells. A synthetic analog of viral dsRNA called polyinosinic-cytidylic acid (poly(I:C)), has been widely used to mimic viral infection. TLR3-deficient (TLR3^−/−^) mice showed reduced pro-inflammatory cytokine production in response to poly (I:C), indicating TLR3 recognizes poly (I:C), more generally dsRNA [31]. Many virus infections are sensed by TLR3, including respiratory syncytial virus (RSV), rhinovirus, reovirus, Epstein-Barr virus (EBV), and herpes simplex virus-2 (HSV2) [32,33,34,35,36]. Moreover, certain DNA viruses like HSV1, HSV2 and EBV, can generate RNA intermediates during viral replication, which can also be sensed by TLR3 [32,36,37,38]. Furthermore, involvement of TLR3 mediated antiviral response has also been studied in HIV-1 and Influenza A virus (IAV) [39,40]. More recently, the role of TLR3 in detection of enterovirus A71 (EV-A71) has been studied, suggesting that expression of TLR3 in HEK293 cells enabled them to sense EV-A71 infection, leading to type I IFN mediated antiviral immunity. The same report also suggests that silencing of TLR3 in mouse and human primary immune cells impaired the activation of IFNβ upon EV-A71 infection, indicating the importance of TLR3 in EV-A71 infection [41]. TLR3^−/−^ mice show lower levels of IFN stimulated genes (ISGs), activated myeloid DCs, and increased viremia against friend retrovirus (FV) infection, suggesting TLR3 mediates antiviral response to FV infection [42]. Moreover, the protective role of TLR3 in HCV and murine coronavirus infection has been studied [43,44]. Several studies have dissected the role of TLR3 in virus infection using TLR3^−/−^ mice with some suggesting that TLR3-dependent recognition of virus is like a ‘double edge sword’. An example is West Nile virus (WNV) infection which induces inflammatory response in TLR3-dependent fashion, which triggers the breakdown of blood brain barrier (BBB), resulting in enhanced brain infection causing lethal encephalitis in TLR3^+/+^ mice [45]. Similarly, the interaction between TLR3 and vaccinia virus (VCV) appears to increase viral replication and to contribute to detrimental effects [46].

TLR7 and TLR8 are phylogenetically close and highly expressed in plasmacytoid DCs (pDCs). This subset of DCs secrete large amount of type I IFN, particularly IFNα in response to viral infection [1,47]. Both TLR7 and TLR8 are involved in sensing of ssRNA viruses, and the recognition is species-specific [48]. Mouse TLR7 and human TLR8 recognize single-stranded-GU rich RNA as natural ligand. Influenza virus, VSV, HIV-1, Sendai virus (SeV), Coxsackievirus B (CBVs), and HCV, are all recognized by TLR7 and TLR8 [49,50,51,52,53].

IFN-α production by pDCs isolated from the bone marrow of TLR7-deficient mice (TLR7^−/−^) was reduced in response to influenza virus or VSV [52]. Moreover, TLR7 facilitates the induction of humoral immunity in response to rabies virus (RABV) which causes fatal encephalitis in mammals [54]. Contrarily, a recent study suggests that TLR7 is involved in sensing of lymph-borne Friend murine leukemia virus (FrMLV), which infects a subtype of B-cells called B-1 cells that permits robust viral replication. This report further suggests that FrMLV exploits innate immune sensing and activation in the B-1 cell population for infection and subsequent spread to other lymphocytes [55].

An in-vivo study reported that in Japanese Encephalitis Virus (JEV) infection in TLR7 deficient (TLR7^−/−^) mice leads to upregulation of TLR8, concluding that TLR8 might play a compensatory role in the regulation of the immune response against JEV infection [56]. Demaria et al. (2010) reported that in the mouse TLR8 is important for the regulation of TLR7, since TLR8 deficiency causes overexpression of TLR7 and a faster NF-κB activation, leading to auto-immunity [57]. Further, a recent study on monocytes suggested that TLR7 and TLR8 activated distinct pathways in RNA virus infections, such as with Coxsackie (CV), encephalomyocarditis (ECMV), IAV, SeV, and VSV [58].

Like TLR7/8, TLR9 is primarily expressed in pDCs and responsible for recognition of bacterial and viral DNA that is rich in unmethylated 2′-deoxyribo (cytidine-phosphate-guanosine) CpG-DNA motifs. The herpes viruses including HSV-1, HSV-2, varicella zoster virus (VZV), cytomegalovirus (CMV), and EBV induce IFN production via TLR9 [59,60,61,62,63,64]. Polymorphism in TLR9 are associated with immune activation and CD4 decline in HIV patients [65]. Additionally, HBV blocks IFN-α production through the specific impairment of TLR9 signaling in pDCs, resulting in establishment of chronic infection [66]. A study in mice found that TLR9 provides protection against enterovirus 71 (EV-71), as the virus induces cellular apoptosis, resulting in tissue damage, the release of endogenous DNA from dead cells and activation of the innate immune system through TLR9 [67].

TLR10 is a pseudogene in mice and the function of TLR10 remains unknown. Some studies suggest that TLR10 is involved in innate immune responses to IAV infection, however this was studied in human peripheral blood monocyte-derived Mɸ [68]. In contrast, others reported that TLR10 acts as a negative regulator of TLR signaling via undefined mechanism [69]. Similarly, another study suggests that TLR10 has a functional role in B-cells. The group created a knock-in mouse model that expresses the full length TLR10 gene under its native human promoter which has the ability to suppress murine B-cell activation, and eventually the adaptive immune response [70]. A lists of TLRs recognizing viruses is shown in Table 1.

### 3.2. Toll-Like Receptor Signaling

The binding of PAMPs to TLRs stimulates two distinct signaling cascades. (i) a MyD88 (myeloid differential primary response)-dependent pathway leading to the production of inflammatory cytokines and chemokines, and (ii) a TRIF (TIR-domain containing adaptor inducing IFN-β)-dependent pathway leading to the production of IFNs and inflammatory cytokines [78]. The MyD88-dependent pathway is used by all TLRs except TLR3, while the TRIF-dependent pathway is triggered by TLR3 or TLR4. Upon ligand stimulation, TLRs recruit a set of adaptor proteins to their TIR domain, including MyD88, MAL (also known as TIRAP), TRIF (also known as TICAM1) and TRAM (also known as TICAM2). Among these, MyD88 and MAL participate in MyD88 dependent signaling, while TRIF and TRAM are only involved in TRIF dependent signaling.

#### 3.2.1. The MyD88-Dependent Pathway

**Figure 1:**
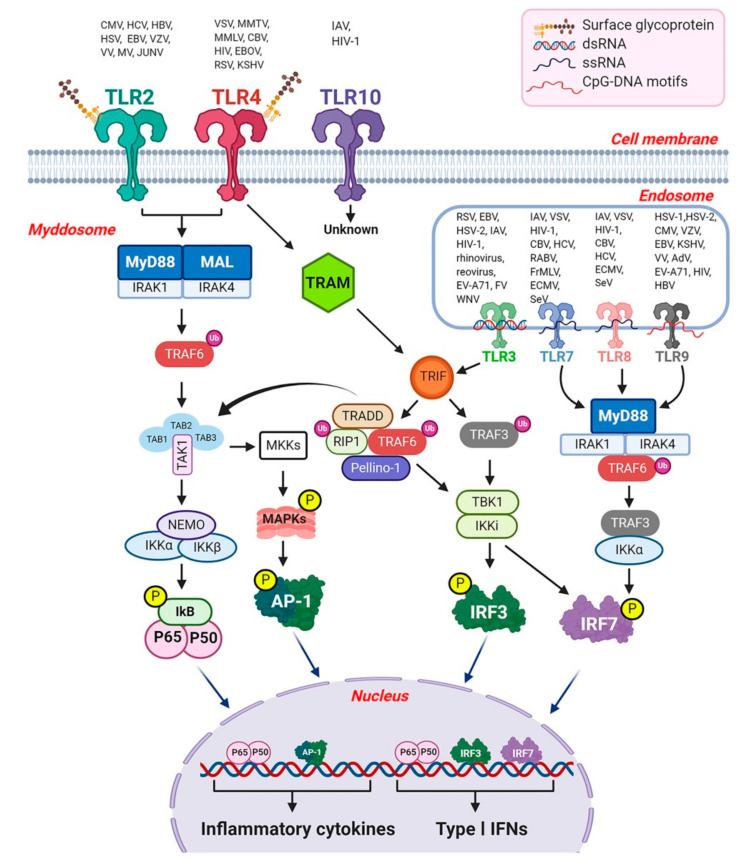
Toll-like receptor (TLR) dependent signaling pathways. TLR 2, -4, -10 are localized to the cell membrane, while TLR3, -7, -8, -9 are localized to the endosomal compartment. Homo- or heterodimerization initiates the signaling via two major downstream adaptor proteins, MyD88 and TRIF. MyD88 dependent signaling is common in all the TLRs except TLR3. However, TLR4 can signal through both MyD88 and TRIF dependent pathway. The TLR engagements results in the formation of myddosome, which consists of MyD88, IRAK1 and IRAK4. In the myddosome, IRAK4 activates IRAK1 which in turn activates TRAF6. TRAF6 then promotes lysine-63 (K63) linked polyubiquitination (Ub) of itself and TAK1. TAK1 forms a complex with the regulatory subunits TAB1, TAB2, and TAB3, which interact with polyubiquitin chains generated by TRAF6 to mediate TAK1 activation. Together this complex phosphorylates the canonical IKK complex, consisting of IKKα and IKKβ and the regulatory subunits NEMO, leading to phosphorylation and subsequent degradation of IκB. This ultimately results in the NF-κB (p65/p50) translocation into the nucleus, and the subsequent transcription of proinflammatory cytokines. MyD88 dependent pathways can lead to the production of type I IFNs, as shown by TLR7 and TLR9 in the endosomal compartment, which requires recruitment of TRAF3 and IKKα to the MyD88-IRAK-TRAF6 complex and subsequent phosphorylation of IRF7 by IRAK1 and IKKα. TLR engagement also induces TRIF activation following TRAF6 and TRAF3 recruitment. TRAF6 recruits RIP1, which activates the TAK1 complex and IKK complex, leading to MAPK, NF-κB, and inflammatory cytokines activation. TRADD is involved in RIP-1 activation. Additionally, Pellino 1 binds to RIP1 to mediate the activation of IKK. TBK1 and IKK complex formation results in the activation and translocation of IRF3 and IRF7 to nucleus, resulting in transcription of type I IFNs. ‘P’ indicates phosphorylation. CMV: Cytomegalovirus; HCV: Hepatitis C virus; HBV: Hepatitis B virus; HSV: Herpes simplex virus; EBV: Epstein-Barr virus; VZV: Varicella zoster virus; VV: Vaccinia virus; MV: Measles virus; JUNV: Junin virus; VSV: Vesicular stomatitis virus; MMLV: Moloney murine leukemia virus; MMTV: Mouse mammary tumor virus; CBV: Coxsackie B virus; HIV: Human immunodeficiency virus; EBOV: Ebola virus; RSV: Respiratory syncytial virus; KSHV: Kaposi sarcoma herpesvirus; IAV: Influenza A virus; HSV-1/-2: Herpes simplex virus -1/2; Rhinovirus; Reovirus; FV: Friend retrovirus; WNV: West Nile virus; RABV: Rabies virus; FrMLV; Friend murine leukemia virus; ECMV: encephalomyocarditis virus; SeV: Sendai virus; EV-A71: Enterovirus A71; VZV: varicella zoster virus (VZV); AdV: Adenovirus; EV-A71: Enterovirus A71.

After TLR activation by PAMPs, TLRs hetero- or homodimerize inducing the recruitment of MyD88 adaptor protein. Next MyD88 forms a complex with IL-1 receptor associated kinase (IRAK) family members (IRAK1 and IRAK4), a complex referred to as the myddosome (Figure 1) [79]. In the myddosome, IRAK4 activates IRAK1, which is then sequentially auto-phosphorylated [80] and released from MyD88 [81]. IRAK1 associates with the RING-domain E3 ubiquitin ligase TRAF6 (tumor necrosis factor (TNF) receptor associated factor 6). TRAF6 then promotes lysine-63 (K63) linked polyubiquitination of itself and the TAK1 (also known as mitogen-activated protein kinase kinase kinase 7, MAP3K7) protein kinase complex with the help of the ubiquitin-conjugating enzymes UBC13 and UEV1A [11]. TAK1 forms a complex with the regulatory subunits TAB1, TAB2, and TAB3, which interact with polyubiquitin chains generated by TRAF6 to mediate TAK1 activation [82]. Together this complex phosphorylates the canonical IKK complex, consisting of IKKα and IKKβ and the regularly subunits NEMO (also called IKKγ), which leads to phosphorylation and subsequent degradation of IκB, ultimately resulting in activation of NF-κB with translocation into the nucleus, and the subsequent release of proinflammatory cytokines [1,11,12]. Importantly, in specific immune cells subsets like pDCs, the MyD88-dependent pathway can lead to the production of type I IFNs, as shown for TLR7 and TLR9 in the endosomal compartment. However, this requires recruitment of TRAF3 (TNF receptor associated factor 3) and IKKα to the MyD88-IRAK-TRAF6 complex and subsequent phosphorylation of IFN regulatory factor 7 (IRF7) by IRAK1 and IKKα [78].

Furthermore, activation of NF-κB and IRF7 is impaired in MyD88-deficient and IRAK4-deficient mice [83,84]. One of reports further suggests that IRAK1 is a specific regulator for TLR7- and TLR9-mediated IFN-α induction in pDCs [83]. In monocytes, IRAK4 activity regulates TAK1 and IKKβ activation, leading to the nuclear translocation of IRF5 and induction of inflammatory cytokines [85]. The MyD88-IRF5 pathway is also used by several TLRs in certain cell types, including Mɸ and conventional DCs (cDCs). Upon TLR ligand stimulation, IRF5 associates with MyD88 complex and translocate into the nucleus, leading to the production of inflammatory cytokines [1,12].

TRAF6 is required for perinatal and postnatal survival of mice, and TRAF6 deficient (TRAF6^−/−^) mice develop osteopetrosis [86]. Further, TRAF6^−/−^ mice were demonstrated to experience 100% mortality within two weeks after birth, and were found to exhibit neural tube defects and exencephaly [87]. Studies have also shown that human parainfluenza virus type 2 (HPIV2) targets TRAF6 and inhibits TRAF6-mediated K63-linked polyubiquitination of IRF7 for the blockade of TLR7 and TLR9 dependent signaling leading to IFN-α production [88], further supporting the critical role of TRAF6.

TAK1 also activates a mitogen-activated protein kinase (MAPK) pathway by phosphorylating MKK3 and MKK6 (members of MAPK family), which subsequently phosphorylate protein kinases such as extracellular signal regulated kinases 1/2 (ERK1/2), c-Jun N terminal kinases (JNK), and p38(p38 mitogen-activated protein kinase), which mediate the activation of AP-1 (activator protein 1) transcription factor family proteins to regulate the production of inflammatory cytokines [1,11,12]. Global knock out of TAK1 in mice is embryonically lethal and it has been widely demonstrated that TAK1 is essential for innate and adaptive immune responses [89,90]. TAK1-deficient cells failed to activate NF-κB and MAP kinases in response to interleukin 1β (IL1β), TNF and TLR ligands [89]. Similarly, reduced phosphorylation of IKKs, p38, and JNK was observed in TAK1 deficient mouse embryonic fibroblast cells (MEFs) after LPS stimulation (Figure 1) [11]. The cell-type specific functions of TAK1 have been reviewed by Ajibade et al. [91]. However, the precise roles of TAB1 and TAB2 are still not clear, since one study showed that they are not essential for the signaling pathways in which TAK1 plays a critical role [92].

#### 3.2.2. The TRIF-Dependent Pathway

TRIF dependent pathways are engaged by TLR3 and TLR4 and result in activation of both IRF3 and NF-κB [1,78]. TLR4 is present on the plasma membrane and signals via MyD88, and it’s trafficking to the endosome is a pre-requisite for the activation of the TRIF-TRAM branch. TLR3 is localized in the endosome and does not require the involvement of TRAM [78]. Upon activation, TRIF interacts with TRAF6 and TRAF3. TRAF6 recruits the adaptor receptor-interacting protein 1 (RIP1), which in turn activates the TAK1 kinase complex and subsequently the IKK complex, mostly in an ubiquitination-dependent manner, leading to activation of NF-κB, MAPKs and inflammatory cytokines (Figure 1). TRAF6 and RIP1 are responsible for activating the NF-κB pathway, and RIP1 undergoes K63-linked poly-ubiquitination after stimulation by TLR3 agonists, and this modification is required for NF-κB activation [12]. Studies have also shown that the adaptor protein tumor necrosis factor receptor type 1-associated DEATH domain (TRADD) is also involved in TRIF-signaling, TRADD binds RIP1, and TRADD-deficient cells show prevention of RIP1 ubiquitination and considerable inhibition of the activation of NF-κB [93], suggesting the involvement of TRADD in RIP1 activation.

Moreover, Pellino-1 is a member of the Pellino family E3 ubiquitin ligases which are also implicated in TLR signaling, and Pellino-1 deficiency in mice display impaired TRIF-dependent NF-κB activation and cytokine production [94], indicating its critical role in TRIF-dependent TLR signals.

TRAF3 recruits the IKK-related kinases TBK1 (TANK [TRAF family member-associated NF-κB activator] binding kinase 1; also known as NAK and T2K) and IKKi (also called IKKε) along with NEMO for IRF3 phosphorylation. This complex subsequently catalyzes phosphorylation of IRF3 and its translocation to the nucleus. TRAF3 is essential for the production of type I IFNs and the innate antiviral response [95]. Myeloid cells from TRAF3 deficient mice showed impaired production of type I IFNs [96]. In addition, the TBK1-IKKi complex can also phosphorylate IRF7, which is considered the master regulator of the innate immune response [97]. IRF7, unlike IRF3, is inducible and expressed at much lower baseline levels. It is regulated on the transcriptional level by a heteromeric complex (IFN stimulated gene factor 3 or ISGF3) which is comprised of signal inducer and activator of transcription 1 (STAT1), STAT2 and IFN regulatory factor 9 (IRF9). Expression of these components drives expression of type I IFNs, which in turn functions, in an autocrine/paracrine fashion, to upregulate these components in a positive feedback loop as well as many antiviral ISGs, including interferon stimulated gene 15 (ISG15) and interferon induced protein with tetratricopeptide repeats 1 (IFIT1). These factors function to prevent viral replication. ISG15 is part of the ubiquitin family while IFIT1 is an abundant short-lived protein that functions by binding 5′ cap viral mRNA and inhibiting eIF4e (eukaryotic translation initiation factor 4E) [98,99].

## 4. C-Type Lectin Receptors (CLR)

CLRs comprise a large family of transmembrane and soluble receptors that contain one or more conserved carbohydrate-recognition domain (CRDs). These CLRs bind carbohydrate moieties in a Ca^2+^-dependent or Ca^2+^-independent manner using CRDs. Hence, the term C-type lectin receptor (CTLR) was introduced for Ca^2+^-independent carbohydrate-binding lectins. Based on their molecular structure and phylogeny the CLRs can be divided into 17 groups. The classification of CLRs has been reviewed in detail by Zelensky and Gready [100].

**Figure 2:**
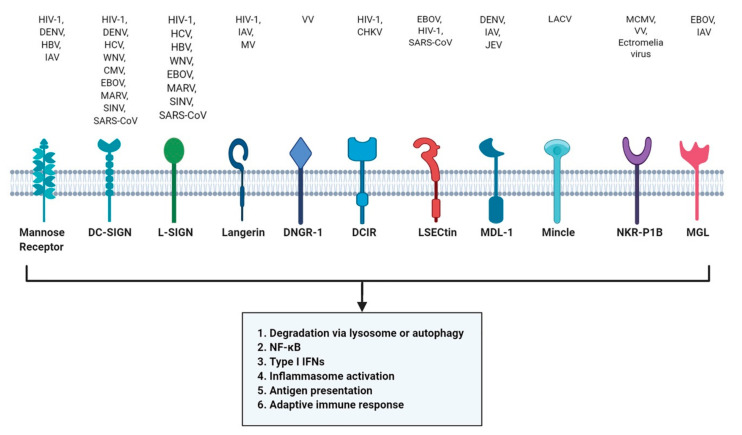
Recognition of viruses by C-type lectin receptors (CLRs). Upon recognition by CLRs, a signaling cascade is activated resulting in autophagy, NF-κB activation, type I IFN production, antigen presentation, inflammasome activation and adaptive immune responses. HIV-1: Human immunodeficiency virus 1; DENV: Dengue virus; HBV: Hepatitis B virus; IAV: Influenza A virus; HCV: Hepatitis C virus; WNV: West Nile virus; CMV: Cytomegalovirus; EBOV: Ebola virus; MARV: Marburg virus; SINV: Sindbis virus; SARS-CoV: Severe acute respiratory syndrome-related coronavirus; MV: Measles virus; VV: Vaccinia virus; CHKV: Chikungunya virus; JEV: Japanese encephalitis virus; LACV: La Crosses virus; MCMV: Mouse cytomegalovirus; Ectromelia virus.

CLRs are expressed in a majority of innate immune cells like DCs, Mɸ, monocytes, Langerhans cells (LCs) and have high affinity for their ligands, including many viruses. The internalization of ligands can have several outcomes dependent on the subtype of CLR and the cell type on which it is expressed. Internalization can result in degradation via lysosome or autophagy [101,102], lead to production of type I IFNs, activation of NF-κB or the inflammasome, or antigen presentation on major histocompatibility complex (MHC) molecules promoting adaptive immune responses [103,104] (Figure 2).

Several studies have shown that CLRs, including mannose receptor (MR) and dendritic cell-specific intercellular adhesion molecule-3-grabbing non-integrin (DC-SIGN), play an important role in virus binding and internalization. Both recognize mannose and fucose structure on the surface of viruses such as HIV-1 and Dengue virus (DENV) [105,106]. HIV-1 can target both MR and DC-SIGN while DENV utilizes MR to evade degradation [106,107]. DC-SIGN also interacts with HCV, Sindbis virus (SINV), WNV, CMV, DENV, SARS-coronavirus, measles virus (MV), IAV and EBOV [103,108].

Lymph node-specific intercellular adhesion molecule-3-grabbing integrin (L-SIGN; also called DC-SIGNR) is a CLR structurally similar to DC-SIGN. Like DC-SIGN, L-SIGN is also exploited by several viruses including HIV-1, EBOV, HCV, HBV, SINV, SARS-CoV, and Marburg virus (MARV) [109,110,111,112,113,114]. Liver and lymph node sinusoidal endothelial cell CLR (LSECtin, or CLEC4G) were found to enhance EBOV and SARS-CoV infection [115]. Another CLR, DAP-12-associating lectin (MDL-1, also known as CLEC5A), mainly expressed by monocytes, MΦ and neutrophils, were recognized by DENV, IAV and JEV [116,117,118].

In contrast, langerin is a type of CLR expressed on LCs and has a role in antiviral protection. The immature LCs can capture HIV-1 via langerin, leading to autophagic degradation of HIV-1 mediated by tripartite motif-containing protein 5 α (TRIM5α), a restriction factor), hence acting as a protective barrier against infection [102,119]. On the other hand, langerin can also function as a receptor for the attachment and dissemination of IAV [120]. Another class of CLR, DC-immunoreceptor (DCIR), can capture HIV-1 and contributes to viral pathogenesis by supporting infection in DCs [121]. In contrast, DNGR-1 or dendritic cell NK lectin group receptor-1 (encoded by CLEC9A) is essential for cross presentation of VCV and the loss of DNGR-1 impairs the CD8^+^ cytotoxic response to VCV hence rendering it unable to facilitate cross-presentation of VCV antigens [122].

A recent study has shown that mincle (also known as CLEC4E) can recognize La Crosse virus (LACV) but has a limited role in antiviral immune response. DCs deficient in mincle (mincle^−/−^) and its adaptor protein caspase activating and recruiting domain 9 (CARD9; CARD9^−/−^) showed reduced amounts of proinflammatory cytokines like IL-6, TNF-α, and no reduction in viral titer [123]. NKR-P1B or killer cell lectin-like receptor subfamily B, member 1 (KLRB1) is transmembrane CTLR that inhibits natural killer (NK) cell function upon interaction with its cognate C-type lectin-related ligand, Clr-b. Mice deficient in NKR-P1B^−/−^ shows that natural killer (NK) cell inhibition by Clr-b was abolished [124]. The mouse cytomegalovirus (MCMV) can target NKR-P1B ligand Clr-b to evade host immune recognition by NK cells [125].

## 5. RIG-I Like Receptors (RLRs)

Retinoic-acid inducible gene I (RIG-I) like receptors (RLRs) are intracellular pattern recognition receptors which function to sense viral RNA. The RLRs family are comprised RIG-I, encoded by the DDX58 gene in the human genome [126,127], MDA5 (melanoma differentiation-associated 5, also known as IFIH1 or Helicard) encoded by the IFIH1 gene [127,128] and LGP2 (laboratory of genetics and physiology 2) encoded by the DHX58 gene [129]. The RLRs recognize viral RNA in the cytoplasm via RNA binding motifs, after which their signaling domain interacts with downstream adapter molecules, resulting in the activation of signaling cascades which lead to the production of type I IFNs, proinflammatory cytokines, and chemokines [130,131].

**Figure 3:**
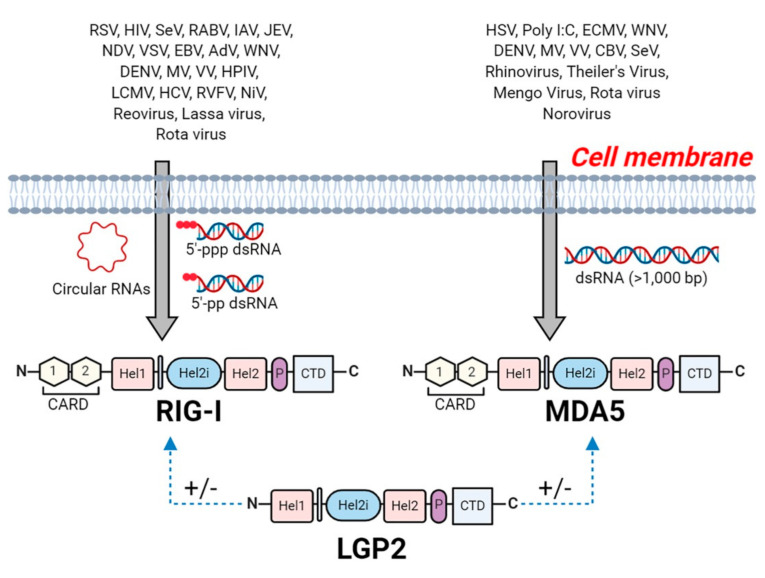
Structure of retinoic acid-inducible gene I (RIG-I)-like receptor, melanoma differentiation-associated gene 5 (MDA5) and laboratory of genetics and physiology 2 (LGP2). Viral detectors, RIG-I and MDA5 share a similar structural pattern: N-terminal region with two caspase activation and recruitment domains (CARDs) and a central DECH-box helicase domain. The helicase domain consists of two Hel-1 and Hel-2, and between them lies a helicase insert domain (Hel2i); the pincer domain and C-terminal domain (CTD). On the other hand, LGP2 lacks the CARD domain and is implicated as a positive or negative regulator of RIG-I and MDA5 (indicated as +/−). RIG-I senses dsRNAs bearing 5′ triphosphate (5′-ppp) moiety or 5 diphosphate (5′-pp) end, and circular RNA (circRNAs); while MDA5 senses long dsRNA (>1000 bp) with no end specificity. RSV: Respiratory syncytial virus; HIV-1: Human immunodeficiency virus 1; SeV: Sendai virus; RABV: Rabies virus; IAV: Influenza A virus; JEV: Japanese encephalitis virus; NDV: Newcastle disease virus; VSV: Vesicular stomatitis virus; EBV: Epstein-Barr virus; AdV: Adenoviruses; DENV: Dengue virus; MV: Measles virus; VV: Vaccinia virus; HPIV: Human parainfluenza virus; LCMV: Lymphocytic choriomeningitis virus; HCV: Hepatitis C virus; RVFV: Rift valley fever virus; NiV: Nipah virus; Reovirus, HSV: Herpes simplex virus; ECMV: Encephalomyocarditis virus; WNV: West Nile virus; CBV: Coxsackie B virus; (Poly(I:C): polyinosinic-cytidylic acid.

RLRs belongs to ATP-dependent DExD/H box RNA helicases family and all three RLRs contain a central DECH-box helicase domain. This domain consists of two RecA like domains (Hel-1 and Hel-2), with a helicase domain known as Hel2i inserted in between [132]. The DECH-box helicase domain enhances the affinity to dsRNA through conformational change and ATP hydrolysis [132,133]. A C-terminal domain (CTD; also known as repressor domain, RD) follows the helicase domain, which is responsible for the recognition of the viral RNAs, including dsRNA and 5′-triphosphate single-stranded RNA (5′ppp-ssRNA) [133]. In addition to the DECH-box helicase domain and CTD, RIG-I and MDA5 consists of two N-terminal CARD domains, which are responsible for activating downstream signaling leading to IRF3 and NF-κB activation [1,132]. However, LGP2 lacks the CARD domain and has therefore been implicated as positive or negative regulator of RIG-I and MDA5 [134,135] (Figure 3).

### 5.1. Recognition of Viral RNA by RLRs

RLR proteins are essential to produce type I IFNs and proinflammatory cytokines against RNA virus infection. Despite structural similarity, RIG-I and MDA5 recognize distinct RNA viruses. RIG-I senses dsRNAs bearing a 5′-ppp moiety [136,137] and/or a 5′ diphosphate (5′-pp) end [138]. Additionally, RIG-I binds to circular RNA (circRNAs), but the molecular structure responsible for this sensing is unknown since circRNAs are single stranded RNA that are joined head to tail and lack phosphate moieties [139]. In contrast, MDA5 senses long dsRNA (>1000 bp) with no end specificity [140].

RIG-I deficient (RIG-I^−/−^) mice were found to be unhealthy and mostly embryonically lethal, while MDA5 deficient (MDA5^−/−^) mice did not show any gross developmental abnormalities until 23 weeks of age [141]. Furthermore, the same report showed that RIG-I is essential to produce IFNs in response to paramyxoviruses, influenza virus and JEV. Cell-specific studies have shown that RIG-I is essential for induction of type I IFNs after infection with RNA viruses in fibroblasts and conventional dendritic cells (cDCs) [142]. This study further indicated that RIG-I is essential in eliciting the immune responses against Newcastle disease virus (NDV), SeV and VSV, in various cells except for pDCs. Additionally, it was reported that some DNA viruses, like EBV and adenoviruses (AdVs), are recognized by RIG-I leading to the production of type I IFNs [143,144]. Samanta et al. reported, that EBV encodes small RNAs (EBERs) which are sensed by RIG-I and activate signaling to induce type I IFNs in EBV-infected cells, however this study utilized plasmid-borne EBERs [144]. Moreover, the same group later reported that EBER induced an anti-inflammatory cytokine IL-10 through RIG-I-mediated IRF-3 but not NF-κB signaling [145]. Interestingly, AdVs encode small noncoding RNAs that are similar to EBV-encoded EBERs, which are recognized by RIG-I [143]. These findings suggest that DNA viruses also activate RIG-I signaling if they produce a small non-coding RNA. Moreover, studies also demonstrated that RNA species generated during the replication of VV are major PAMPs able to activate RIG-I or MDA5 dependent IFNβ and pro-inflammatory cytokines production, however, the use of PRRs for VV depends on cell type and cell state. This study also indicated that these RNA species can induce activation of apoptosis in PKR (a type of CDS)-dependent manner [146]. Another study suggested that a modified VV Ankara (MVA; an attenuated dsDNA poxvirus) can induce an up-regulation of RIG-I, MDA-5 and IPS-1, but only MDA5 and IPS-1 were found to mediate the production of IFNβ and IFNβ-dependent chemokines in macrophages, indicating the critical role of MDA5 [147]. Although Cheng et al. reported in 2007 that both dsDNA and dsRNA share common pathway to trigger innate antiviral defense response in human cells, dsDNA triggers that pathway upstream of the dsRNA-interacting protein RIG-I [148].

Single nucleotide polymorphisms (SNPs) in IFIH1/MDA5 have been linked to autoimmune disorders, type 1 diabetes (T1D), psoriasis, rheumatoid arthritis, vitiligo, multiple sclerosis (MS), and Systemic Lupus Erythematosus (SLE) [149,150,151,152,153,154,155]. Additionally, mutations in IFIH1/MDA5 are associated with Singleton-Merten Syndrome and with Aicardi-Goutières syndrome [156,157]. MDA5 is critical for picornavirus detection [141]. However, it was later reported that polio virus, a member of *Picornaviridae* family, induced cleavage of MDA5 to subvert the type I IFNs production in response to viral infection, concluding that not all members of picornavirus family are sensed by MDA5 [158]. IFIH1 (gene encoding for MDA5) knock out mice demonstrated that MDA5 is essential for the type I IFNs response to poly (I:C) and ECMV [159]. Interestingly, induction of type I IFNs by Theiler’s virus and Mengo virus, which also belong to the picornavirus family is mediated by MDA5 [141]. Moreover, MDA5 senses murine norovirus [160]. Recent human studies have shown that children with inherited MDA5 deficiency or IFIH1 mutation are more susceptible to viral infections [161,162,163].

Some viruses including WNV and DENV are recognized by both RIG-I and MDA5 [164,165,166]. In vitro studies have shown that both RIG-I and MDA5 can sense and respond to poly (I:C) [167]. Furthermore, both RIG-I^−/−^ and MDA5^−/−^ mice are highly susceptible to infection with RNA viruses. Moreover, the membrane (M) protein of SARS-CoV-2 inhibits type I and III IFN production by targeting RIG-I/MDA5 signaling. Mechanistically, the SARS-CoV-2 M protein prevents RIG-I-MAVS, MAVS-TBK1, TRAF3-TBK1 interactions ultimately impeding the phosphorylation and nuclear translocation of IRF3 [168]. In vitro studies on SARS-CoV-1 have indicated that RIG-I and MDA5 are transcribed during the infection, although it is not clear if the virus is sensed by RIG-I/MDA5 [169]. Similarly, mouse coronavirus is recognized by MDA5 in brain Mφ/microglia and induces the production of type I IFN [170]. Others reported that mouse coronavirus is sensed by both RIG-I and MDA5 in oligodendrocytes cells [171]. On the other hand, studies in mice models have reported the involvement of TLR3 in response to SARS-CoV-1 infection [172,173]. Table 2 summarizes viruses recognized by RLRs.

### 5.2. RLR Signaling

**Figure 4:**
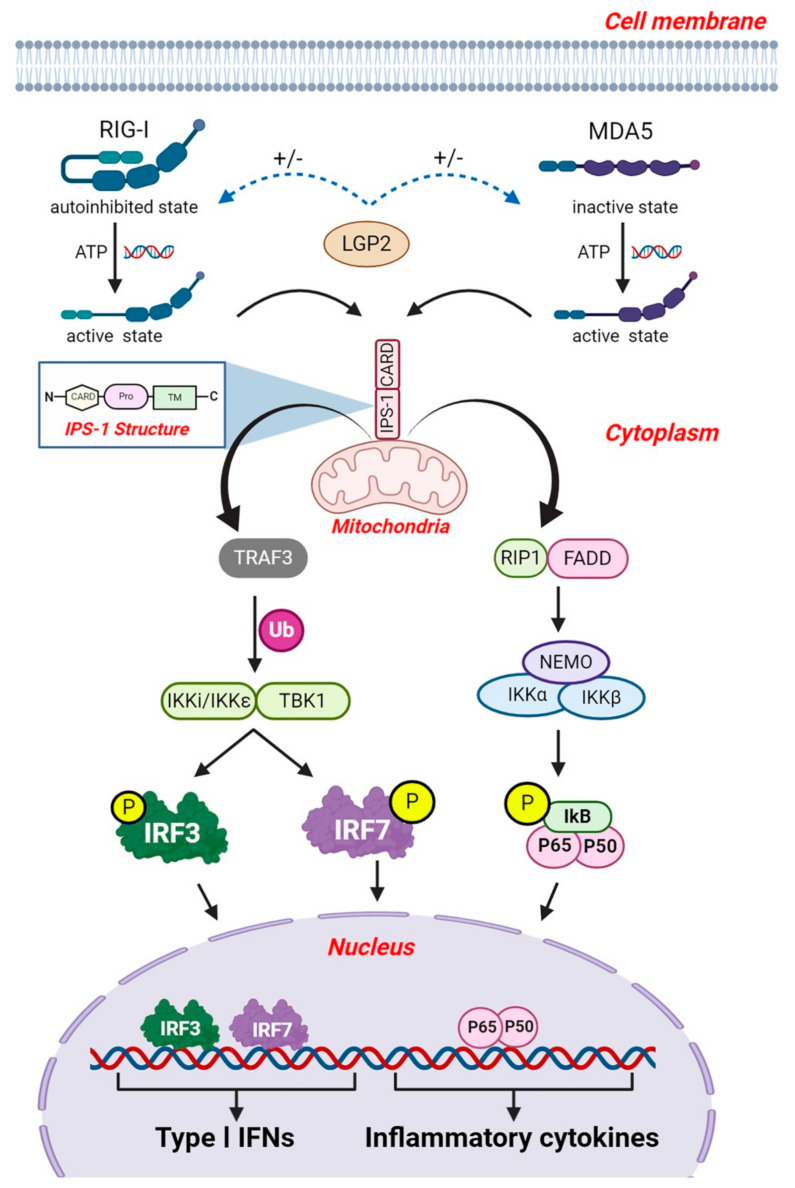
RLR signaling pathway. During viral infection, PAMPs in the cytoplasm are recognized by RIG-I and MDA5 leading to their activation. RIG-I and MDA5 interact with IPS-1 adaptor protein located on the mitochondria via its CARD domain. This interaction results in downstream activation of TRAF3, followed by the K63 polyubiquitination (Ub) of TBK1 and IKK*i*/IKKε, which phosphorylates IRF3 and IRF7, leading to the production of type I IFNs. IPS-1 also activates NF-κB via FADD and RIP1 dependent pathways. FADD and RIP1 form a complex followed by the recruitment of IKKα and IKKβ. This transcription factor complex mediates the translocation of NF-κB (p65/p50) into the nucleus, which initiates the production of inflammatory cytokine. ‘P’ indicates phosphorylation.

RLRs (RIG-I and MDA5) interact with the CARD domain of IFNβ promoter stimulator-1 (IPS-1, also known as mitochondrial antiviral-signaling protein or MAVS, virus induced signaling adaptor protein (VISA) and CARD adaptor inducing IFNβ or CARDIF). IPS-1 molecules are located on the outer membrane of mitochondria, peroxisomes, and the ER where they serve as the essential adaptor protein for RLR signal transduction [7]. Structurally, IPS-1 consists of a N-terminal CARD like domain that associated with the RIG-I and MDA5, proline rich region, and a transmembrane domain on C-terminal (Figure 4) [174]. IPS-1 signaling is necessary for type-I IFNs production in most cell types and polymorphisms in IPS-1 are linked to type I IFNs deficiency in humans [175]. IPS-1-deficient (IPS-1^−/−^) mice show severe defects in both RIG-I and MDA5 mediated induction of type I IFNs and inflammatory cytokines and were susceptible to RNA virus infection [174]. Furthermore, IPS-1^−/−^ mice are more susceptible to acute infection with pneumonia virus of mice (PVM) and WNV [176,177]. Additional studies suggest that IPS-1 signaling contributes to the recruitment of pDCs and type I IFN production, which plays a critical role in effective antiviral responses [176,177,178]. All these studies support an essential role of IPS-1 in the RLRs signaling. Moreover, transgenic mice expressing human CD150, an entry receptor for MV, serve as mouse model for MV infection. Studies utilizing these transgenic mice revealed that CD150^+^/MAVS^−/−^ mice were not permissive to MV infection in vivo due to the substantial production of type I IFN. The MyD88-dependent type I IFN production in cDCs and pDCs result in initial protection against MV, inducing an antiviral state in neighboring cells of the initial target cell, suggesting the involvement of MyD88-dependent and MAVS-independent signaling for the production of type I IFNs during MV infection [179].

**Table 2:** Viruses recognized by Retinoic-acid inducible gene I like receptors or RLRs.

PRRs/Receptor	Localization	Adapter	Viruses PAMPS	Viruses	Reference
RIG-I	Cytoplasm	IPS-1	5′ppp dsRNA, 5′pp dsRNA, Circular RNA, ssRNA, dsRNA, Virus-encoded RNA	Newcastle disease virus	[142]
				West Nile Virus	[164]
				Sendai virus	[142]
				Vesicular stomatitis virus	[142]
				Epstein-Barr virus	[144]
				Adenoviruses	[143]
				Dengue virus	[165]
				Influenza A virus	[141]
				Japanese encephalitis virus	[141]
				Rabies Virus	[180]
				Ebola virus	[181,182]
				Nipah Virus	[182]
				Lassa Virus	[182]
				Rift valley fever virus	[182]
				Rota virus	[183]
				Measles virus	[184]
				Vaccinia virus	[146]
				Human immunodeficiency virus	[185]
				Human parainfluenza virus	[186]
				Hepatitis C virus	[187]
				Reovirus	[188]
				Lymphocytic choriomeningitis virus	[189]
MDA5	Cytoplasm	IPS-1	dsRNA, RNA (>1000 bp)	Encephalomyocarditis picornavirus	[159]
				Theiler’s virus	[141]
				Mengo virus	[141]
				Murine norovirus	[160]
				West Nile virus	[164]
				Dengue virus	[165]
				Sendai virus	[190]
				Rotavirus	[183]
				Measles virus	[184]
				Vaccinia virus	[146]
				Coxsackie B virus	[191]
				Herpes simplex virus	[192]
				Rhinovirus	[163]

The IPS-1 and RIG-I/MDA5 interaction results in downstream activation of TRAF3, which catalyzes its own K63 poly-ubiquitination followed by the recruitment of TBK1 and IκB kinase-ε (also known as IKK*i*/IKKε), which phosphorylates IRF3 and IRF7 [131]. TRAF3 is essential for activation of TBK1/IKKε, given that several deubiquitinases, including ubiquitin thioesterase OTUB1/B2, deubiquitinating enzyme A (DUBA), and NmrA-like family domain-containing protein 1 or NMRAL1 (HSCARG), have been shown to downregulate RLR-mediated type I IFNs production by removing K63-linked poly-ubiquitin chains from TRAF3 or TRAF6 [193,194,195]. Moreover, mice carrying whole-body inducible deletion of TRAF3 were sensitive to VSV infection [196]. The same report further suggests that TRAF3 is essential for production of type I IFNs in mouse embryonic fibroblasts (MEFs) but not in MΦ implying its cell type- and stimulus-specific role. After phosphorylation, IRF-3/-7 homodimers and/or heterodimers translocate into the nucleus and bind to ISREs, resulting in the expression of type I IFNs [1,7]. IFNs subsequently induce the expression of ISGs. ISGs have antiviral and immune-modulatory actions that serve to regulate the onset and actions of the adaptive immune responses. Deletion of both IRF3 and IRF7 in mice resulted in the absence of induction of IFNα and IFNβ subsequently causing a wider spread of IAV in lungs [197]. These knock out mice showed increase in granulocyte infiltration and reduced activation of the adaptive immune response. In addition, knockout of either IRF3 or IRF7 indicated a crucial role of IRF7 as the driver of IFNβ expression in an infection model of DENV [198].

The RLR signaling pathway also activates the transcription factor NF-κB via FAS-associated death domain-containing protein (FADD) and RIP1 dependent signaling. IPS-1 interacts with FADD and RIP1 via its non-CARD region to facilitate NF-κB activation (Figure 4) [199]. Together FADD and RIP1 form a molecular complex, followed by the recruitment of IKKα and IKKβ [1,7]. This transcription factor complex mediates translocation of NF-κB into the nucleus and results in production of type I IFNs and inflammatory cytokines [1,199,200]. FADD and RIP1 are crucial for signaling against viruses, since cells lacking FADD and RIP1 were defective in production of type I IFNs and were more susceptible to viral infection [201].

LGP2 is essential to produce antiviral responses but lacks a CARD domain and thus fails to autonomously transduce signaling [202]. Structural and functional studies show that LGP2 promotes the nucleation of MDA5 oligomerization on dsRNA and signaling [135]. LGP2 also potentially co-cooperates with other PRRs to regulate IFN signaling. LGP2 was originally suggested to be a negative inhibitor of RIG-I and MDA5 [167]. However, recent studies showed LGP2 positively regulates the RLR signaling particularly MDA5-mediated IFN signaling [134,203,204,205]. Moreover, LGP2 deficient mice (LGP^−/−^) were highly susceptible to infection with EMCV [134]. LGP2 also plays an essential role in IFN responses induced by HCV infection by promoting MDA5 recognition of HCV RNA in hepatocytes [206]. Altogether, the exact role of LPG2 in IFN signaling is incompletely understood and remains controversial.

## 6. NOD-Like Receptors

Like RIG, the NOD-like receptors (NLRs) are intracellular cytosolic sensors. They are expressed in many immune cells, including lymphocytes, macrophages, DCs and also in non-immune cells, like in epithelial cells [207]. Structurally, NLR proteins are comprised of three domains, a centrally located NOD or nucleotide binding (NBD) domain (also known as NACHT), C-terminal leucine-rich repeats (LRRs), and a variable N-terminal interaction domain. The N-terminal domain consist of CARD, pyrin domain (PYD), acidic transactivating domain or baculovirus inhibitor repeats (BIRs) [207]. The NACHT domain is critical for activation and mediates ATP-dependent self-oligomerization. The LRR region senses PAMPs, while the N-terminal domain is responsible for homotypic protein-protein interaction [6,207]. Based on N-terminal domains, NLRs are classified into four subfamilies: the acidic transactivation domain (NLRA), the baculoviral inhibitory repeat-like domain (NLRB), the caspase activation and recruitment domain (CARD; NLRC), and the pyrin domain (NLRP) [208]. Based on phylogenetic analysis NLRs can be divided into three subfamilies: the NOD, the NLRPs (also called NALPs or nucleotide-binding oligomerization domain, leucine-rich repeat and pyrin domain containing) and the IPAF (also called NLR family CARD domain-containing protein 4 or NLRC4) [6]. The complete classification of NLRs is described elsewhere in the literature [6,208]. NOD1 and NOD2 (also known as NLRC1 and NLRC2) are the first identified NLRs involved in detection of bacterial components. Upon pathogen recognition, NLRs activate the NF-κB complex, leading to expression of pro-inflammatory and chemotactic cytokines [209].

NLRPs and IPAF subfamilies are involved in the formation of the inflammasome. The best characterized is the NLRP3 inflammasomes which is activated by some fungi, bacteria and viruses, such as SeV, AdVs, WNV, and influenza virus [210,211,212]. In this article we will focus on NLRP3 as it is the only member of the NLR family known to provide innate immunity against viruses.

## 7. The NLRP3 Inflammasome

**Figure 5:**
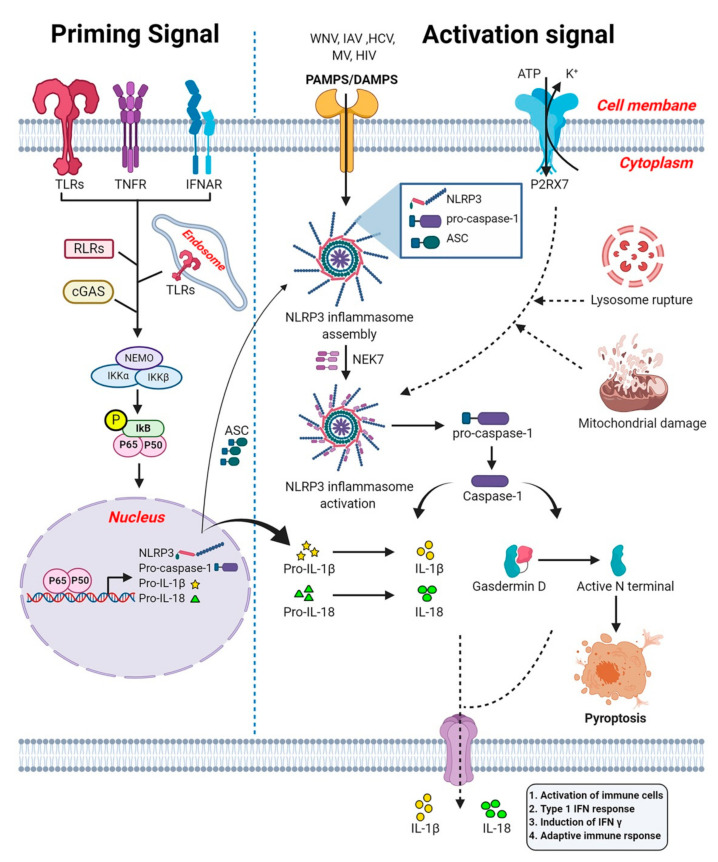
NLRP3 Inflammasomes signaling pathway. A two-step signal for NLRP3 inflammasome activation. The priming signal (left panel) is induced by PRRs, IFNAR or TNFR activation that further leads to the activation of NF-κB (p65/p50) and its subsequent translocation to the nucleus where it triggers the expression of NLRP3, pro-caspase-1, pro-IL-1β, and pro-IL-18. Following the priming step, an adaptor protein (ASC), recruits NLRP3 and pro-Caspase-1, forming a complex called the NLRP3 inflammasome (right panel; activation signal). Next, activation of NLRP3 triggered by multiple signals including PAMPs/DAMPS, disruption of potassium homeostasis, lysosomal rupture, mitochondrial damage and a mitotic factor NEK7, leads to the activation of Caspase-1. Activated caspase-1 induces secretion of proinflammatory cytokines IL-1β and IL-18 and regulates pyroptosis, an inflammatory programmed cell death. Capasase-1 activates propyroptotic factor gasdermin D, which is responsible for forming pores on the membrane of infected cells, facilitating the release of IL-1β/IL-18 and inducing pyroptosis. ‘P’ indicates phosphorylation. WNV: West Nile virus; IAV: Influenza A virus; HCV: Hepatitis C virus; MV: Measles virus; HIV: Human immunodeficiency virus.

NLRP3 (also known as NALP3 and cryopyrin) is activated by two step signaling: (i) a priming step and (ii) an activation step (Figure 5). The priming step is induced by PRRs, Type I IFN receptor (IFNAR) or TNFα receptor (TNFR) activation that further leads to the activation of NF-κB and its subsequent translocation to the nucleus where it triggers the expression of NLRP3, pro-capase-1, pro-IL-1β, and pro-IL-18 [213]. The NLRP3 activation step is triggered plethora of stimuli such as PAMPs, DAMPs, lysosomal rupture, or mitochondrial damage [213,214] Upon activation, NLRP3 leads to the oligomerization and recruitment of adaptor protein ASC (apoptosis-associated speck-like protein containing a CARD domain or PYCARD). The ASC contains PYD and CARD domain. The pyrin domain of NLRP3 binds to the PYD domain of ASC via PYD-PYD interaction. The CARD domain of adaptor protein ASC recruits pro-caspase-1 and these protein-protein interactions form a complex called the NLRP3 inflammasome [215]. The NLRP3 activation also requires the mitotic NIMA-related kinase 7 (NEK7) which bridges adjacent NLRP3 subunits to mediate the activation of the NLRP3 inflammasome [216]. As the next step, the NLRP3 inflammasome triggers the autocleavage of pro-caspase-1 and formation of active caspase-1 [217,218]. Caspase-1 is important for the proteolytic processing of the pro-inflammatory cytokines IL-1β and IL-18 and the pro-pyroptotic factor gasdermin D (GSDMD) [208]. GSDMD is responsible for forming pores on the membrane of infected cells, facilitating the release of IL-1β/IL-18 and inducing pyroptosis [219].

IL-1β has several functions including activation of immune cells and help in the recruitment of activated immune cells, such as neutrophils to the inflammatory site to aid in the elimination of invading viruses. Both IL-1β and IL-18 are also responsible for the initiation of the adaptive immune response [220,221]. Altogether, the NLRP3 inflammasome contributes to the antiviral response, for example by promoting host protective responses during influenza and WNV infection [212,222].

Mutations in the NLRP3 gene are known to cause an autoinflammatory disease called cryopyrin-associated periodic syndrome (CAPS). In addition, aberrant NLRP3 inflammasome activation can also lead to severe pathological injuries. The NLRP3 inflammasome has been shown to be involved with the pathogenesis of Mayaro virus [223]. In addition, IL-1β production by NLRP3 inflammasome is a central feature of liver inflammation during HCV infection [224]. Hence, the modulation of NLRP3 inflammasome activity is important and can provide promising therapeutic avenues for the intervention in viral diseases.

## 8. Cyclic GMP-AMP Synthase

**Figure 6:**
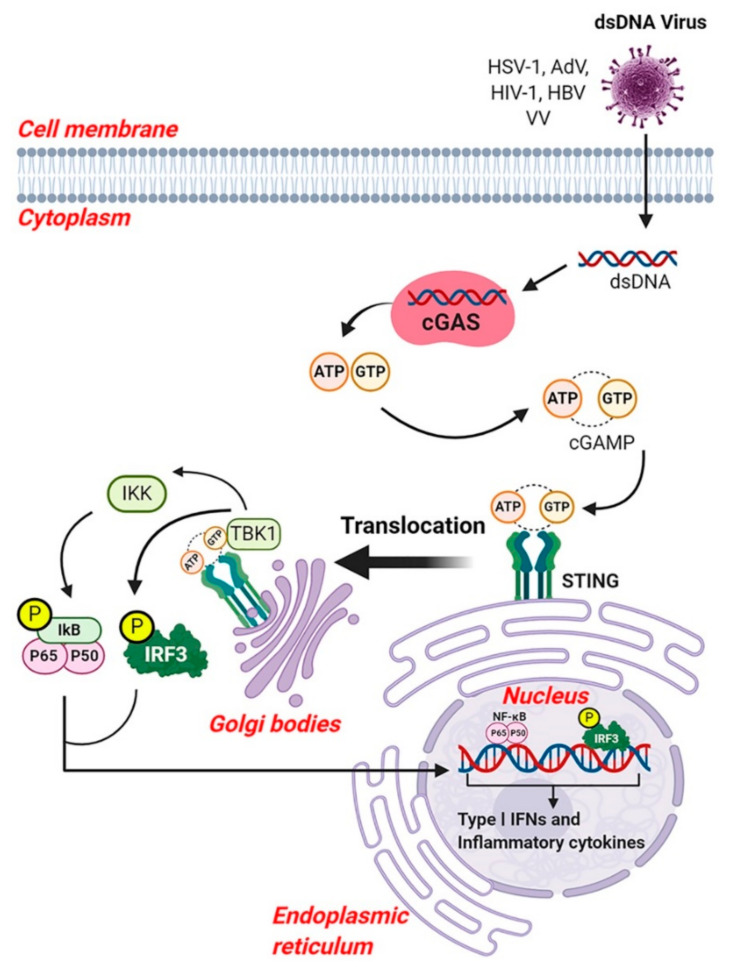
The cGAS-STING signaling pathway. Cyclic GMP-AMP synthase (cGAS) are cytosolic DNA sensors, capable of sensing dsDNA. After recognition of dsDNA, cGAS catalyzes the formation of cyclic GMP-AMP (cGAMP), which in turn binds to stimulator of IFN genes (STING), located on the endoplasmic reticulum membrane. The binding of cGAMP to STING promotes the translocation of STING to Golgi bodies. During translocation, STING triggers the activation of TBK1 and IKK complexes. This complex then causes the phosphorylation of IRF3 and NF-κB. The active IRF3 and NF-κB (p65/p50) translocate into the nucleus and activate the transcription of type I IFNs and other inflammatory cytokines. ‘P’ indicates phosphorylation. AdV: Adenovirus; HBV: Hepatitis B virus; HIV-1: Human immunodeficiency virus 1; HSV-1: Herpes simplex virus 1; VV: Vaccinia virus.

Cyclic guanosine monophosphate-adenosine monophosphate (GMP-AMP) synthase (also known as cGAS, cGAMP synthase) is a cytosolic DNA sensor that plays an important role in production of type I IFNs. CyclicGAS belongs to the nucleotidyl-transferase enzyme family, which plays a major role in cGAS-stimulator of IFN genes (STING) sensing pathway which is capable of recognizing dsDNA [225]. After sensing dsDNA, cGAS catalyzes the formation of cyclic GMP-AMP (cGAMP), which in turn binds to STING and triggers its cellular trafficking and activation of TBK1 and IKK complexes. TBK1 causes the phosphorylation of IRF3, and the active IRF3 dimer then translocates into the nucleus and triggers the production of type I IFNs. The phosphorylated IKK complex releases NF-κB for translocation into the nucleus where it induces transcription. Both IRF3 and NF-κB are essential for the production of type I IFNs and other inflammatory cytokines (Figure 6) [225,226].

DNA viruses, such as HSV-1, AdVs and modified VV can trigger cGAMP production leading to activation of IFNs via STING [226,227,228]. Knockdown of cGAS, STING or TBK1 in cells results in reduction of activated IRF3, IRF3 responsive genes, STAT1 and STAT2 following exposure AdVs [228]. VV can replicate its DNA genome in the cytoplasm of infected cells and can suppress the innate immune response through the expression of multiple inhibitors acting upstream and downstream of the immune pathway, and thus block cytosolic DNA sensing, which was recently reviewed by El-Jesr et.al 2020 [229]. The host’s DNA sensing of VV is complex and hence an attenuated VV strain, such as MVA or genetically engineered mice is widely used to gain insights into VV immunity. However, a limitation is that DNA sensing in mice differs from humans [230,231]. Several studies indicated that type I IFN production by MVA is dependent of cGAS and its downstream adaptor STING [227,232]. Mice or human cells deficient in cGAS or STING show impaired production of type I IFNs in response to MVA [229,233]. Moreover, cGAS-deficient mice showed higher viral titer and greater susceptibility to VV (western reserve strain) [234]. These studies suggested a crucial role for CDS in the immune response to VV infection. In addition, cGAS is capable of sensing DNA produced during reverse transcription by retrovirus such as HIV-1 [235,236]. Interestingly, some RNA viruses with no DNA intermediate in their life cycle, also trigger cGAS for immune defense via unknown mechanisms [237,238]. Another study suggests, HBV infection suppresses cGAS expression and function in humanized mice, indicating a mechanism by which HBV evades cGAS sensing [239].

## 9. Interferons during Viral Infection

Interactions of viruses with PAMPs and PRRs usually lead to the production of IFNs. IFNs are signaling proteins (cytokines) released from host cells and characterized by their ability to interfere with viral replication [240]. These cytokines are released in response to the pathogen invasion or inflammation and used to communicate between cells, in a paracrine and autocrine fashion, to trigger a protective immune defense mechanism in the host [241]. IFNs are also critical since they affect both innate and adaptive immune responses [242,243]. Based on their receptors, IFNs are classified as three major groups (i) IFN type I family (IFN-I) encompasses 13 IFNα subtypes in humans (14 in mice), IFNβ, IFNω, IFNκ, and IFNɛ, (ii) IFN type II (IFN-II) encodes for IFNγ, (iii) IFN type III (IFN-III) encodes for IFNλ1, IFNλ2, IFNλ3, and IFNλ4 [242,244]. Type I IFNs have potent antiviral activity. Upon viral stimulation, the infected cells begin to produce these IFNs which function in an autocrine and paracrine fashion and induce an anti-viral state that is characterized by the expression of distinct ISGs. IFNs interfere with viral replication and simultaneously alert immune cells, such as natural killer (NK) cells and MΦ and other neighboring uninfected cells. The uninfected cell induces the production of anti-viral proteins that inhibit, interfere or degrade viral nucleic acids and enhance antigen presentation by increasing MHC class II expression in antigen-presenting cells [241].

## 10. PRR in Activation of Adaptive Immune Response

Several studies have been performed to dissect the role of PRRs in shaping the adaptive immune response. Briefly, PRRs induce different innate immune response as discussed, the activation of adaptive immune response primarily depends on the ability of PRR to induce DCs. DCs bind and take up antigens, become activated, upregulate MHC and co-stimulatory molecules and migrate to the local lymphoid tissues to interact with naive T cells that are specific to the microbial antigen [8]. DCs process the pathogen by phagocytosis, and present antigens in association with MHC molecules to T-cells [245]. We have discussed in this review the role of several PRRs in activation of DCs. NK cells are also involved in adaptive immune response, since they cross talk with DC in several ways, including killing of immature DCs and promoting DC maturation, which leads to overall enhanced antigen presentation to T cell [246]. Similarly, macrophages also function as antigen presenting cells, thus contributing to the activation of T helper cell [230].

## 11. Consequences of the Innate Anti-Viral Immune Response in the CNS

The cellular processes involved in innate immunity occur rapidly as a means to prevent viral infections from expanding. However, if the innate immune response fails to alleviate the infection or if the viral infection evades the immune system, an acute infection cannot be contained. The infection can become chronic and/or spread into other tissues, including the CNS. The persistent infection in the CNS results in inflammation of distinct anatomical regions including the meninges (meningitis), brain (encephalitis) and spinal cord (myelitis) caused by release of an assortment of pro-inflammatory cytokines and other deleterious factors [247,248,249]. This release of cytokines can be massive, constituting what is called a ‘cytokine storm’ of the innate immune response in the CNS resulting in neuropathology and consequently behavioral and neurologic deficits [250]. Generally, viruses enter the organism in the periphery, including neurotropic viruses, and if the innate immune response is insufficient, they can migrate into the CNS via a multitude of routes.

### 11.1. Viral Entry into the CNS

**Figure 7:**
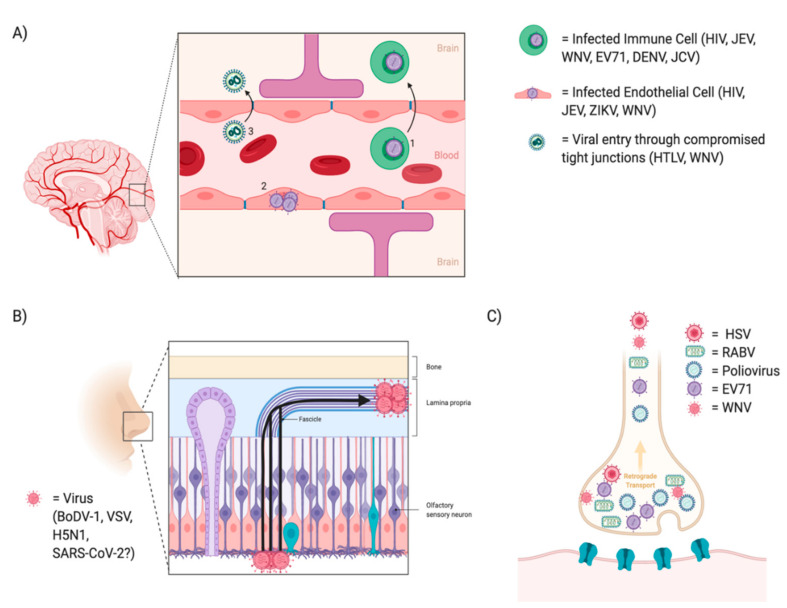
Mechanisms of viral entry into the central nervous system. (**A**) Viral entry through the BBB can occur via 3 methods: (1) The Trojan Horse model where a virus leverages a permissive host immune cells as a vehicle, (2) direct infection of brain microvascular endothelial cells (BMVECs) and (3) viral entry through dysregulation of tight junction proteins. (**B**) Viral entry through the nasal epithelium occurs through infection of the peripherally located olfactory receptors neurons and into the lamina propria which ultimately terminates in the olfactory bulb. (**C**) Infection through peripheral nerves allows for viruses to move in a retrograde manner towards multiple brain centers. HIV: Human immunodeficiency virus; JEV: Japanese encephalitis virus; WNV: West Nile virus; EV71: Enterovirus A71; DENV: Dengue virus; ZIKV: Zika virus; HTLV: Human T Lymphotropic Virus.

The nervous system is partitioned into the peripheral nervous system (PNS) and the CNS. The PNS is much more easily accessible to viral infection spread as the peripheral nerves are in direct contact with an assortment of tissues. On the other hand, the CNS is a partially protected system consisting of a multi-layered physical barrier as well as specialized cell types involved in immune recognition. This protection serves to defend resident cells from infection and damage [251]. However, neurotropic viruses, including HSV, RABV, DENV, WNV, Zika Virus (ZIKV), JEV, EV-71, Human Polyomavirus 2 (JCV), Poliovirus, Borna Disease Virus (BoDV), Avian Influenza A (H5N1), VSV, CMV, HIV, Human T Lymphotropic Virus (HTLV)-1 and recently SARS-CoV-2 have a diverse set of routes to enter the CNS: (1) the circulating infected host peripheral cells or free virus can enter the brain parenchyma by transmigrating through or breaching the blood brain barrier (BBB), (2) viruses can infect neurons in the nasal olfactory epithelium and spread via axonal transport along the olfactory nerve into the CNS and finally (3) direct transmission from peripheral nerves into the spinal cord via motor or sensory neurons (Figure 7) [250,252].

#### 11.1.1. Infection through the Blood Brain Barrier

The BBB is a protective barrier lining the vasculature of the CNS. It is comprised of brain microvascular endothelial cells (BMVECs) which are interconnected by tight junctions whose role is to regulate entry into the CNS. Along the perimeter of the BBB lie astrocytes and pericytes. These cells give structure and molecular support and form what is called the neurovascular unit [253]. During infections, the BBB becomes permissive to immune extravasation which serves to traffic peripheral immune cells into the CNS for immune support; however, viruses can leverage this as a transport vehicle into the CNS. This is the first form of entry through the BBB called the “Trojan horse” model [254]. HIV in particular infects peripheral monocyte/macrophages and lymphocytes, which express CD4 and chemokine receptors, as essential mediators of HIV viral entry into cells [255].

In particular infected monocyte/macrophages extravasate into tissues, including the brain parenchyma where they appear to be perpetuating a condition known as HIV-associated neurocognitive disorder (HAND). This is of global concern as 37 million people worldwide are diagnosed with HIV, and an estimated 20–30% of people living with HIV develop HAND [256,257]. The neurocognitive deficits are likely mediated by inflammation induced by macrophage/microglia, rather than viral infection of neurons [255]. In addition to its route into the CNS, a recent paper suggested that an HIV reservoir in the brain can egress back into the periphery, in part due to trafficking of infected CD4^+^ T cells out of the brain [258]. The egressed virus evolved at a rate and pattern similar to an acute peripheral infection. This study highlights the dynamic flow of neurotropic viruses into the CNS as well as the ramifications of egression of these neurotropic viruses back to the periphery.

JEV is another virus that leverages this trojan horse method as it has been shown to replicate in lymphocytes and macrophages, making these host immune cells potential carriers of JEV into the CNS [259,260,261]. This method of extravasating into the CNS via phagocytic host cells is also seen in WNV. Specifically, WNV has been shown to increase the expression of cell adhesion molecules (VCAM-1 and E-selectin), which are often associated with both an increased influx of infected host immune cell and increased viral load in the CNS [262,263]. Additionally, CMV has been shown to invade the CNS via hematogenous spread and replicate in brain parenchyma [264]. In fact, in a mouse model of CMV, after intraperitoneal inoculation free virus and cell associated virus were both detected, indicating neuroinvasion by host cell permissiveness to the BBB or through free virus crossing the BBB [264]. CMV shows no cell tropism in the CNS as microglia, astrocytes, oligodendroglial cells, neurons and brain microvascular endothelial cells can all be infected, although CMV-positive astrocytes are the hallmark of a productive infection [265]. Other viruses that extravasate using host immune cells include EV-71 [266], DENV [267] and JCV [268].

Trafficking via infected host immune cells is not the only means of neuroinvasion, infection and activation of specialized endothelial cells (i.e., BMVECs) provide a second means of viral spread. For example, membrane lipid rafts and heparan sulfate present on permissive cells, including BMVECs, may play a role in the neuroinvasion of HIV-1 [269]. JEV, similar to HIV, can invade the CNS through endothelial cells. Elegant electron-microscopic techniques were used to determine that JEV is endocytosed by cerebral blood vessels and breaches the BBB, where it is transported across the capillary endothelium and pericytes via vesicles [270]. Blockage of heparan sulfate, a factor located on endothelial cells of the BBB, by bovine lactoferrin is also able to inhibit JEV [271]. Beyond HIV and JEV, other viruses including arthropod-borne ZIKV, DENV and WNV have also been shown to persistently infect and replicate in BMVECs and are subsequently released basolaterally towards neuronal compartments [263,267,272,273]. WNV infected BMVECs reach peak replication in the cytoplasm at 2 days post infection as shown by staining with anti-WNV envelope antibodies as well as by WNV titers in the supernatants of infected cells by plaque forming assays [263]. ZIKV infection of BMVECs resulted in increased type I and III IFN secretion and inflammatory cytokines (IL6 and CCL5) as well as a release of infectious virus particles; however, it did not increase endothelial permeability or BBB disruption in vitro or in vivo [274].

A third scenario permitting neuroinvasion involves the dysregulation of the tight junction proteins interconnecting the BMVECs. One study suggests that infection with HTLV-1 significantly reduced expression of the tight junction protein ZO-1 allowing for BBB permeability and lymphocyte passage through the monolayer [275]. Additionally, WNV, which is mosquito-borne and causes encephalitis, can invade the CNS via many pathways including dysregulation of endothelial tight junctions potentially mediated by an increase in MMP-9 levels [276]. Studies using an MMP-9 knocking out mouse model have indicated its critical role in WNV neuroinvasion as levels of brain viral load, infiltrating leukocytes and inflammatory cytokines were decreased as compared to WT mice [276]. Additionally, a study infecting BMVECs with a neurovirulent strain of WNV (NY99) showed an increase in mRNA and protein expression of cell adhesion molecules VCAM and E-selectin concomitantly with peak WNV replication [263]. Type I IFNs, IFNα and IFNβ, play a direct role in tightening tight junction formation, increasing barrier integrity and decreasing transendothelial trafficking of WNV. The mechanism by which this process occurs involves a decrease in TNFα and IL-1β, which are factors known to induce endothelial cell breakdown and tight junction dysregulation via modulation of Rho GTPase signaling [277].

#### 11.1.2. Infection through the Nasal Epithelium

Nasal epithelium provides a transit for viral infection into the CNS. Olfactory receptor neurons have projections that begin in the olfactory epithelium (periphery) and traverse through the cribriform plate to terminate in the olfactory bulb (CNS). Here in the olfactory bulb they synapse onto glomeruli with dendrites of mitral cells, these mitral cells then project broadly into the parenchyma including regions such as the limbic system (i.e., amygdala and hippocampus) and entorhinal cortex, which is a direct relay station into the frontal cortex [278]. This route of entry has been noted for several neurotropic viruses including HSV-1, BoDV-1, H5N1, VSV and potentially SARS-CoV-2 [278].

BoDV-1 results in a fatal encephalitis in humans by infection of olfactory receptor neurons. In vivo studies show the presence of BoDV-1 genomic RNA in adult and juvenile olfactory receptor neurons 7 days post infection [279]. Additionally, H5N1 has been shown to invade the CNS by the intranasal route in a human child with severe immune deficiency [280]. Here viral antigen was detected in the olfactory bulb, olfactory tract and gyrus rectus. Similarly, in a mouse model, VSV was shown to infect olfactory receptor neurons and 12–24 h post infection VSV antigens were found in the olfactory epithelium and lamina propria [281]. However, VSV does not travel along the trigeminal nerve to enter the CNS, unlike some other viruses.

In a retrospective study of patients in Wuhan, patients with severe SARS-CoV-2 infection had notably more neurological manifestations compared to patients with non-severe infection. This included acute cerebrovascular disease (5.7% vs. 0.8%), impaired consciousness (14.8% vs. 2.4%) and skeletal muscle injury (19.3% vs. 4.8%) [282]. It is clear the virus enters the CNS as there is expression of SARS-CoV-2 RNA in the CSF of patients as well as neuropathological manifestations indicated above [283]. The route of viral entry into the CNS is unclear for SARS-CoV-2; however, it is likely via two routes (1) droplets carrying the virus may find an entry through the cribriform plate and (2) potentially by hematogenous spread and blood brain barrier penetration [284]. Following entry into the CNS, SARS-CoV-2 requires the presence of angiotensin-converting enzyme 2 (ACE2) as receptor, which is critical for cellular entry. Interestingly, a subset of neurons expressed ACE2, and using an iPSC-derived brain sphere model, SARS-CoV-2 was able to infect a fraction of neural cells [285]. Viral particles were found in both the cell body as well as neurites [285]. In addition, other receptors including neuropilin-1, may be involved in facilitating entry of SARS-CoV-2 into the brain. Neuropilin-1 is expressed in the olfactory epithelium, as well as other olfactory related regions including olfactory tubercles and para-olfactory gyri [286]. The olfactory epithelium seems to be the strongest candidate as a mechanism for neuroinvasion, as another study has identified goblet cells and ciliated cells of the nasal epithelium as being high expressors of ACE2 [287].

#### 11.1.3. Infection through Peripheral Nerves

Peripheral nerves, including the neuromuscular junction (NMJ), provide a transit for viral infections into the spinal cord and towards different brain centers. The prototypical example of infection through the NMJ is the zoonotic RABV, which is primarily spread through rabid animal bites or by contamination of scratch wounds by virus infected saliva [288]. RABV replicates in the inoculated striated and connective tissue, infecting myocytes, before traversing in a retrograde fashion up peripheral nerves at the neuromuscular junction [288]. The nicotinic acetylcholine receptor (nAChR), the neuronal cell adhesion molecule (NCAM), and the p75 neurotrophin receptor (p75NTR) may all be target receptors for the rabies virus [289]. Live cell imaging studies have shown that RABV and p75NTR can internalize together and are transported in acidic compartments, although at different rates, indicating the ability of rabies to hijack this transport system and even manipulate it [290]. RABV receptors are located on the motor endplate, while sensory and autonomic endings are unaffected [291]. Another virus, poliovirus, exhibits a similar infection of the spinal cord, although through a different route of transmission. Poliovirus is transmitted primarily via fecal-oral spread through the oropharynx, making it a highly communicable disease primarily in densely-populated areas with poor sanitation [292]. Poliovirus infects the mucosal epithelial cells, which eventually follow peripheral neurons into the CNS [293]. Polio viral antigens can be found in the neuronal cell bodies at the anterior horn of the spinal cord, among other locations including the tonsils, lymph nodes of the neck and Peyer’s patches. The infection ultimately manifests as poliomyelitis and paralysis as the anterior horn is the location for motor output [292,293]. Poliovirus acts on the CD155 receptor, primarily located on axonal membranes and then migrates in a retrograde manner towards other brain centers, including motor neurons of the thalamus, the hypothalamus and in the bulbar form of poliomyelitis a fatal involvement of the brain stem can be seen [292,294]. However, poliovirus can enter the CNS through a CD155 independent mechanism as well, which is seen as hematogenous spread of poliovirus crossing the BBB at a high rate [295]. WNV, as mentioned previously has multiple routes of neuroinvasion, and it has been shown using neuronal cultures and through inoculation of the sciatic nerve that WNV spreads in both a retrograde and anterograde direction via axonal transport [296]. Axonal transport of WNV resulted in spinal cord infection, neuronal injury and acute flaccid paralysis, and that spread occurred via extracellular secretion from axons [296]. In addition, another neurotropic virus, EV-71, showed an inflammatory profile consisting of CD68 positive macrophage/microglia and CD8 positive T cells in the gray matter of the spinal cord, as well as brainstem, hypothalamus and subthalamic and dentate nuclei [297]. Other neurotropic viruses that have been reported to reach the CNS through peripheral nerves also include HSV-1 and HSV-2 [298].

### 11.2. Innate Immune Response in the CNS

The innate immune response in the CNS includes the response of resident cells to viral infection of brain tissue (i.e., microglia and astrocytic responses) as well as resident CNS innate cells dynamically interacting with trafficking T cells entering from the periphery [299]. Beyond this, peripheral cells of the innate system (neutrophils, peripheral monocyte/macrophages etc.) can migrate into the CNS upon viral infection after receiving chemokine cues. In addition, from a molecular basis, many of the signaling pathways and PRRs, including TLRs and RIG-I, that exist in the periphery maintain relevance in the CNS. Although neuroinflammation is a necessity to alleviate damage from viral infection, it is a double-edged sword where excessive inflammation can be deleterious. Therefore, it is a constant push and pull by the host to regulate these inflammatory processes.

#### 11.2.1. Microglia and Astrocytes

The innate immune response in the CNS differs from that of the periphery primarily based on a unique set of resident cell types: microglia and astrocytes. Based on fate mapping studies, microglia are embryonically derived from primitive myeloid progenitors originating from the yolk sac [300,301]. Microglia migrate into the brain parenchyma where they have several functions, including phagocytosis, release of antiviral components and synaptic pruning. One of their functions includes the sensing of extracellular ATP, which is released from virally infected neurons [302]. Detection of ATP by P2Y12 and P2X7 receptors on the surface of microglia cue the cells to migrate to the virally infected neuron and induce antiviral as well as phagocytic activity [303]. Microglia also express PRRs, which ultimately induce a downstream type I IFN response through a cascade of intracellular signaling involving components MAVS, TRIF, STING, IRF3/7, STAT1, all of which were discussed above [5]. This type I IFN response has been shown to be anti-inflammatory and neuroprotective in a chronic infection/inflammatory setting. For example, Type I IFN receptor (IFNAR) expression in the glomeruli of the olfactory bulb is crucial in prevention of the spread of virus through the nasal epithelium and into the brain parenchyma [304]. In addition, upon intranasal VSV infection, IFNβ was produced by the neuroectodermal cells (primarily astrocytes, and to a lesser extent neurons) and found to be protective of other IFNβ negative distal brain regions leading to a containment of viral spread [305]. To corroborate this study, another study injected VSV into the caudate-putamen, and linked a type I IFN response to limiting viral spread at the site of inoculation, as well as inhibiting the spread to neurons in networks connected by synapses [306]. STING, a player in the canonical signaling for initiating type I IFN production against cytosolic DNA, plays a critical role in controlling CNS infection. STING knockout mice expressed increased neuropathological signs, as well as increased viral load and dissemination in the CNS of WNV infected mice compared to WT [307].

#### 11.2.2. Neurons

Neurons possess the ability to host an innate immune response as well. For example, in WNV infection the granule cell neurons of the cerebellum express a host defense pathway gene signature including STAT1 and IFN dependent genes [308]. Neurons express many PRR pathways that induce anti-viral responses. Among them is the NOD-like receptor NLRP3 which is capable of suppressing WNV replication in neurons via IL-1β production [212,309]. Additionally, TLR3 is required for induction of type I IFN in WNV infected cortical neurons [310]. RABV infections regulate other PRR players by activating viral detector RIG-I at the mRNA and protein level, shown in vitro by RABV infection of two neuronal cell lines (NT2N and SK-N-SH) [311]. In ZIKV, the clinical manifestation of microcephaly is thought to occur due to infection and apoptosis of neural progenitor cells [312]. Studies using human embryonic stem cell-derived cerebral organoids to recapitulate early-stage brain development implicated TLR3 activation to neural progenitor depletion [313]. Endosomal TLR7, RIG-I/MDA5 and cGas-STING also contribute to sensing of ZIKV pathogen signals [314]. MV, the etiological agent of measles, is another virus which can induce a potent cell-mediated, overall protective immune response, as seen with T cell activation of IFNα and IL-2 [315]. MV, however, can also induce multiple forms of severe encephalitis including primary measles encephalitis, acute postinfectious measles encephalomyelitis, measles inclusion body encephalitis and the rare but almost always fatal subacute sclerosing panencephalitis (SSPE) [315,316]. MV can infect neurons and induce a chronic, inflammatory immune response including the production of CXCL10 and CCL5 [317]. Chemokines CXCL10 and CCL5 colocalized with measles virus infected neurons based on immunofluorescent imaging of infected mouse brain sections. Additionally, ablation of chemokines with neutralizing antibodies inhibited the T cell trafficking to the CNS by 20–50%, indicating a neuron specific antiviral response to help recruit T cells to the CNS [317].

Although the processes of viral sensing remain conserved between the periphery and the CNS, it is possible the expression and robustness of the responses varies between peripheral cells (i.e., macrophages, pDCs) and CNS cells (i.e., microglia, neurons, astrocytes).

### 11.3. Cytokine and Chemokines Regulating the BBB

The release of proinflammatory cytokines/chemokines during the innate immune response provides triggers to help regulate the tightness and permissiveness of the BBB as well as assist in trafficking peripheral immune cells into the CNS. The release of cytokines including TNFα, IL-1β and type I IFNs can differentially regulate the permeability of the BBB. Increased TNFα and IL-1β expression compromise the tight junction proteins, claudin 5 and ZO-1, located between the BMVECs, allowing the BBB to be more permissive [318]. This effect on the BBB is mediated by expression of MMP-9 in response to TNFα induced activation of p21-activated-kinase-1 (PAK1). Conversely, the loss of type I IFN specific signaling in cerebellar astrocytes promoted BBB permeability to the hindbrain in a WNV infection model [319]. Viral entry increased specifically in the hindbrain, however, viral replication and tropism remained the same further indicating type I IFN’s role specifically in modulating permeability at the level of the BBB [319].

### 11.4. Human Inborn Errors of Innate Immune Pathways in the CNS

Maternal exposure to viral pathogens, including VZV, HSV, ZIKV and HIV, can cause fetal brain damage including microcephaly, white matter disease, cerebral atrophy and calcification [312,320]. Human inborn errors in select genes can underlie type I interferonopathies, for example Aicardi-Goutières syndrome is characterized by genetic mutations in three-prime-repair exonuclease 1 (TREX1), SAM and HD domain-containing deoxynucleoside triphosphate triphosphohydrolase 1 (SAMHD1) and interferon-induced with helicase C domain 1 (IFIH1) among others [321]. Recently, a set of loss of function recessive mutations in ubiquitin-specific peptidase 18 (USP18), a key negative regulator of type I interferon response, have been associated grossly with brain calcification and polymicrogyria [320]. Post-mortem fetal brain pathology in patients showed positive staining of phosphorylated STAT1, upregulation of MHC class II identifying an inflammatory state. Cellularly, USP18-deficient patient fibroblasts, exhibited an exacerbated type I interferon response of IFIT1, MX2 and ISG15 effector genes following IFNα treatment [320]. Another example, biallelic loss of functions mutations in gene DBR1, (encoding RNA lariat debranching enzyme) which functions in hydrolyzing 2′-to 5′ branches phosphodiester bonds of intron RNA, led to impaired HSV-1 viral infection in the brainstem in a TLR3 independent manner [322]. DBR1 is ubiquitously expressed, but most prominently in the spinal cord and brainstem and seems to be implicated in HSV-1 infection of the brainstem, which occurs in 5% of patients with HSV-1 encephalitis [323,324]. Gain of function mutations have been described for another important ISG inducer, STAT1, in patients with JC virus-induced progressive multifocal leukoencephalopathy (PML), including a novel L400Q mutation [325]. A more comprehensive review of human inborn errors on the innate immune response has been published by Bucciol et al. [326].

### 11.5. Viral Evasion of the Innate Immune Response

Viruses have developed multiple approaches to hampering the antiviral innate immune response which have been discussed in recent reviews [322,323,327]. Therefore, we discuss here only a few aspects of this important topic. One primary mechanism involves the ability of the virus to target PRRs and the downstream signaling molecules that induce the type I IFN response. For example, in the context of HIV infection, microglia show no significant degree of IRF3 activation, but rather a decrease in IRF3, indicating a mechanism by which HIV is capable of limiting IFNβ production [328]. Mechanistically, this may in part be due to the ability of HIV accessory protein, Vpu, to proteolytically cleave IRF3 via caspases [324]. Beyond IRF3, HIV can downregulate TRAF6 and VISA expression resulting in enhanced viral replication in macrophages, including those infiltrating the CNS during HIV infection [325]. Another example is the A188V mutation in the NS1 gene of a strain of ZIKV which is capable of binding TBK1 and inhibiting its phosphorylation [320]. Phosphorylated TBK1 is crucial for the phosphorylation and activation of IRF3, which translocates to the nucleus and drives transcription of type I IFN genes and in the case of ZIKV specifically debilitates IFNβ induction. Other ZIKV nonstructural proteins including NS2A, NS2B, NS4A and NS5 can prevent IFNβ induction by restricting components in the RIG-I pathway including TBK1, IKKe and/or IRF3 [320]. RIG-I sensing also seems to be essential for initial viral detection and controlling of WNV [321]. WNV induces IRF3 activation approximately 12–16 h post infection, in contrast to an assortment of other viruses which induce IRF3 within 3–10 h post infection suggesting WNV delays initiation of the host response [321]. RIG-I null mouse embryo fibroblasts showed an increase in WNV replication compared to wild type mouse embryo fibroblasts [321]. Therefore, therapeutic approaches that involve exogenously stimulating the type I interferon response and drawing viruses out of latency are being employed.

## 12. Therapeutic Approaches to Viral Infections of the CNS

The current standard of care for viral infections of the CNS is supportive care and antiviral drugs [329]. However, treating viral infections of the CNS remains challenging since even cocktails of drugs are often ineffective at completely depleting the viral reservoir, such as in HIV infection [255,330,331]. Additionally, antiviral drugs are frequently unable to cross the BBB leading to persistence of a viral load specifically in the CNS [332]. To this end, methods leveraging the different routes of CNS invasion as well as augmenting the host innate immune system can be used to diminish viral entry and reduce viral load within the CNS.

One methodology is to inhibit the entry of a virus through its avenues into the CNS. In HIV-1, a potential entry point is through cell associated heparan sulfate proteoglycans (HSPGs) a surface moiety, found on permissive BMVECs of the BBB. One study highlighted that enzymatic digestion of HSPGs dramatically inhibited HIV-1 infection as determined by the common p24 infection assay [269]. HSPG inhibition, as well as neuropilin 1 and Glut1, also prevented the infection of endothelial cells by HTLV-1 [275]. These findings suggest the therapeutic potential of targeting HSPGs for the prevention of CNS infection by retroviruses.

As described previously, IFNβ is an integral component of the innate immune system to alert neighboring cells of viral threat. IFNβ is an already FDA approved treatment for the neurodegenerative disease multiple sclerosis [333]. Although the mechanism of IFNβ mediated protection in MS is not completely understood, key studies have given us insight into potential mechanisms. One possibility involves the ability for IFNβ to at least temporarily inhibit the opening of the BBB in relapsing-remitting MS patients [334]. IFNβ is able to help localize tight junction proteins, including ZO-1 and ZO-2, after an inflammatory insult presumably to prevent peripheral T cell recruitment into the CNS [335]. Additionally, serum from IFNβ treated relapsing-remitting MS patients has shown a significant increase in IL-10, an anti-inflammatory cytokine which functions in prevent Th1 polarization of T cells [336]. In vivo studies have also shown that intranasal administration of recombinant IFNβ can alleviate much of the neuronal damage seen in NeuroHIV. Administration of mouse recombinant IFNβ rescued neuronal dendrites and synapses in an HIVgp120 transgenic mouse model that recapitulates the neuronal and behavioral deficits of NeuroHIV and HAND [337]. In vitro studies using mouse mixed cerebrocortical cultures have also implicated IFNβ in gp120 induced neuroprotection [337]. Although the molecular mechanisms underlying IFNβ’s role in NeuroHIV are only partially known, studies have suggested that the beneficial effect is linked to its ability to induce neuroprotective β-chemokines CCL3, -4, -5, which restrict HIV infection and provide neuroprotection [337,338]. Addition of CCL4, -5 to fetal human microglia has been shown to decrease HIV viral load [326]. Furthermore, CCL4 or CCL5 has been shown to protect rat cerebrocortical neurons against neurotoxicity of HIVgp120 in an AKT-dependent manner [339]. It remains to be shown, if these β-chemokines provide protection to the brain in infections by viruses other than HIV-1.

In the case of HSV infection, a combination of acyclovir and IFNα shows approximately a 30% reduction in mortality after HSV challenge compared to acyclovir alone [340]. Mouse models have also highlighted the importance of TLR9 activation 24 h prior to infection helped in inducing a type I IFN response when challenged with HSV encephalitis [341]. Interestingly, studies from the same group indicated that TLR9 antagonist administration resulted in an improved outcome when administered after HSV challenge [342]. This is likely due to taming of the pro-inflammatory cytokine release experienced immediately after infection.

The various cellular mechanisms of viral sensing are essentially the same in both the periphery and the CNS. However, the consequences of interferon and cytokine production in response to viral infection are frequently more severe in the brain than in the periphery. On the other hand, a tightly controlled antiviral response that avoids overt inflammation and cell death permits viruses to establish reservoirs in the CNS.

## Figures and Tables

**Table 1:** Viruses recognized by toll like receptors or TLRs.

PRRs/Receptor	Localization	Adapter	Viruses PAMPS	Viruses	Reference
TLR2	Cell membrane	MyD88	Envelope glycoprotein, core protein	Cytomegalovirus	[14]
				Hepatitis C virus	[13]
				Hepatitis B virus	[15]
				Herpes simplex virus	[16]
				Measles virus	[71]
				Varicella-zoster virus	[72]
				Epstein-Barr virus	[73]
				Vaccinia virus	[74]
				Junin virus	[18]
TLR3	Endosome	TRIF	dsRNA, RNA, siRNAs, self RNA, polyI:C	Respiratory syncytial virus	[33]
				Rhinovirus	[35]
				Reovirus	[31]
				Herpes simplex virus-2	[36]
				Influenza A virus	[40]
				Enterovirus A71	[41]
				Friend retrovirus	[42]
				West Nile virus	[45]
				Murine coronavirus	[44]
				Human immunodeficiency virus	[39]
				Epstein-Barr virus	[32]
				Hepatitis C virus	[43]
TLR4	Cell membrane	MyD88 and TRIF	Glycoprotein, envelop protein, fusion protein,	Vesicular stomatitis virus	[19]
				Ebola virus	[20]
				Mouse mammary tumor virus	[21]
				Moloney murine leukemia virus	[21]
				Coxsackie B virus	[75]
				Respiratory syncytial virus	[76]
				Human immunodeficiency virus	[22]
TLR7/8	Endosome	MyD88	ssRNA	Vesicular stomatitis virus	[52]
				Human immunodeficiency virus	[51]
				Coxsackie B virus	[49]
				Hepatitis C virus	[53]
				Sendai virus	[50]
				Influenza virus	[52]
				Rabies virus	[54]
				Friend murine leukemia virus	[55]
TLR9	Endosome	MyD88	Viral DNA	Herpes simplex virus 1	[64]
				Herpes simplex virus 2	[63]
				Varicella-zoster virus	[62]
				Cytomegalovirus	[59]
				Epstein-Barr virus	[61]
				Human immunodeficiency virus	[65]
				Hepatitis B virus	[66]
				Enterovirus 71	[67]
TLR10	Cell membrane	Unknown	Envelope protein, RNA	Influenza A virus	[68]
				Human immunodeficiency virus	[77]

## Data Availability

Not applicable.

## Abbreviations

AIDS: Acquired immune deficiency syndrome; AP-1: Activator protein 1; AdV: Adenoviruses; ACE2: angiotensin-converting enzyme 2; BBB: Blood brain barrier; BIR: Baculovirus inhibitor repeats; BMVECs: Brain microvascular endothelial cells; BoDV: Borna Disease Virus; CDS: Cytosolic DNA sensors; CMV: Cytomegalovirus; CNS: Central nervous system; CV: Coxsackie; CBVs: Coxsackievirus B; CRDs: Carbohydrate-recognition domains; CTLR or CLR: C-type lectin receptor; CTD: C-terminal domain; CARD: Caspase activating and recruiting domains; circRNAs: Circular RNA; cDCs: Conventional dendritic cells; CARD9: Caspase activation and recruitment domain 9; cGAMP or GMP-AMP: Cyclic guanosine monoposphase-adenosine monophosphate synthase; cGAS: Cyclic GMP-AMP synthase; CSF: Cerebrospinal fluid; CpG-DNA motifs: cytidine-phosphate-guanosine-DNA motifs; CAPS: Cryopyrin-associated periodic syndrome; dsRNA: Double stranded RNA; DAMPs: Damage-associated molecular patterns; DUBA: Deubiquitinating enzyme A; DENV: Dengue virus; DCs: Dendritic cells; DCIR: DC-immunoreceptor; DC-SIGN: Dendritic cell specific intercellular adhesion molecule-3-grabbing non- integrin; EBV: Epstein-Barr virus; EV-A71: Enterovirus A71; EBOV: Ebola virus; ER: Endoplasmic reticulum; EMCV: Encephalomyocarditis virus; EBERs: EBV encodes small RNA; ERK1/2: Extracellular signal regulated kinases ½; FADD: FAS-associated death domain-containing protein; FV: Friend retrovirus; FrMLV: Lymph-borne Friend murine leukemia virus; GSDMD: Gasdermin D; HCV: Hepatitis C virus; HBV: Hepatitis B virus; HSV: Herpes simplex virus; HSV-1: Herpes simplex virus-1; HSV2: Herpes simplex virus-2; HSPs: Heat shock proteins; HMGB1: High mobility group box-1 protein; HPIV2: Human parainfluenza virus type 2; HIV: Human Immunodeficiency Virus; HAND: HIV Associated Neurocognitive Disorder; HIV: Human Immunodeficiency Virus; HTLV-1: Human T Lymphotropic Virus; HAND: HIV Associated Neurocognitive Disorder; HSPGs, Heparan sulfate proteoglycans; HPIV: Human parainfluenza virus; IAV: Influenza A virus; IFNs: Interferons; IFIH1: IFN-induced with helicase C domain 1; IFIT1: Interferon induced protein with tetratricopeptide repeats 1; IFNAR1: Type I interferon receptor; IL1-β: Interleukin 1beta; IPS-1: IFNβ promoter stimulator-1; IRF: Interferon response factor; ISGs: Interferon stimulated genes; ISG15: Interferon stimulated gene 15; ISGF3: Interferon stimulated gene factor 3; JCV: Human Polyomavirus 2; JEV: Japanese encephalitis virus; JNK: c-Jun N terminal kinase; JUNV: Junin virus; KSHV: Kaposi sarcoma herpesvirus; LPS: Lipopolysaccharide; LCs: Langerhans cells; LACV: La Crosses virus; LCMV: Lymphocytic choriomeningitis virus; LRR: C-terminal leucine-rich repeats; LSECtin: Liver and lymph node sinusoidal endothelial cell C-type lectin receptor; L-SIGN: Lymph node-specific intercellular adhesion molecule-3-grabbing integrin; LGP2: Laboratory of genetics and physiology 2; MMTV: Mouse mammary tumor virus; MMLV: Moloney murine leukemia virus; MDA5: Melanoma differentiation-associated 5; MHC: Major histocompatibility complex; MR: Mannose receptor; MCMV: Mouse cytomegalovirus; MARV: Marburg virus; MyD88: Myeloid differential primary response dependent pathway; MEFs: Mouse embryonic fibroblast cells; Mɸ: Macrophages; MAVS: Mitochondrial antiviral-signaling protein; MMP-9: Metalloproteinase 9; MV: Measles virus; MS: Multiple sclerosis; NK: Natural killer cells; NDV: Newcastle disease virus; NBD: Nucleotide binding domain; NLRs: NOD-like receptors; NCAM: Neuronal cell adhesion molecule; NJM: Neuromuscular junction; PVM: Pneumonia virus of mice; PYD: Pyrin domain; PAMPs: Pathogen-associated molecular patterns; polyI:C: Polyinosinic acid-cytidylic acid; pDCs: Plasmacytoid dendritic cells; PRR: Pattern recognition receptors; PNS: Peripheral nervous system; PAK1: p21-activated-kinase-1; P75NTR: p75 neurotrophin receptor; RLRs: Retinoic acid-inducible gene-I-like receptors; RD: Repressor domain; RABV: Rabies virus; RIG-I: Retinoic-acid inducible gene I; RIP1: Receptor-interacting protein 1; RSV: Respiratory syncytial virus; RVFV: Rift valley fever virus; SAMHD1: SAM and HD domain containing deoxynucleoside triphosphate triphosphohydrolase 1; SARS-CoV-2: severe acute respiratory syndrome coronavirus 2; SINV: Sindbis virus; siRNA: Small interfering RNAs; SeV: Sendai virus; SNP: Single nucleotide polymorphisms; STING: Stimulator of interferon genes; ssRNA: Single stranded RNA viruses; SeV: Sendai virus; STAT1: Signal inducer and activator of transcription 1; SLE: Systemic Lupus Erythematosus; T1D: Type 1 diabetes; TNF: Tumor necrosis factor; TRIF: TIR-domain containing adaptor inducing interferon (IFN)-β; TLRs: Toll-like receptors TBK1: TANK (TRAF family member-associated NFKB activator)-binding kinase 1; TRAF6: tumor necrosis factor (TNF) receptor associated factor 6; TRIM5α: Tripartite motif-containing protein 5 α; TRADD: Tumor necrosis factor receptor type 1-associated DEATH domain; TREX1: three-prime-repair exonuclease 1; VISA: virus induced signaling adaptor protein; VZV: Varicella zoster virus; VSV: Vesicular stomatitis virus; VV: Vaccinia virus; WNV: West Nile virus; ZIKV: Zika Virus.
